# Sub-national tailoring of malaria interventions in Mainland Tanzania: simulation of the impact of strata-specific intervention combinations using modelling

**DOI:** 10.1186/s12936-022-04099-5

**Published:** 2022-03-17

**Authors:** Manuela Runge, Sumaiyya G. Thawer, Fabrizio Molteni, Frank Chacky, Sigsbert Mkude, Renata Mandike, Robert W. Snow, Christian Lengeler, Ally Mohamed, Emilie Pothin

**Affiliations:** 1 Swiss Tropical and Public Health Institute, Basel, Switzerland; 2 University of Basel, Basel, Switzerland; 3 National Malaria Control Programme, Dodoma, Tanzania; 4 Ministry of Health, Community Development, Gender, Elderly, and Children, Dodoma, Tanzania; 5 Centre for Tropical Medicine and Global Health, Nuffield Department of Clinical Medicine, University of Oxford, Oxford, UK; 6 Population Health Unit, Kenya Medical Research Institute-Wellcome Trust Research Programme, Nairobi, Kenya; 7 CHAI, Clinton Health Access Initiative, New York, USA

**Keywords:** Malaria, Strategic planning, Mathematical modelling, OpenMalaria, Tanzania, Stratification, Intervention allocation

## Abstract

**Background:**

To accelerate progress against malaria in high burden countries, a strategic reorientation of resources at the sub-national level is needed. This paper describes how mathematical modelling was used in mainland Tanzania to support the strategic revision that followed the mid-term review of the 2015–2020 national malaria strategic plan (NMSP) and the epidemiological risk stratification at the council level in 2018.

**Methods:**

Intervention mixes, selected by the National Malaria Control Programme, were simulated for each malaria risk strata per council. Intervention mixes included combinations of insecticide-treated bed nets (ITN), indoor residual spraying, larval source management, and intermittent preventive therapies for school children (IPTsc). Effective case management was either based on estimates from the malaria indicator survey in 2016 or set to a hypothetical target of 85%. A previously calibrated mathematical model in OpenMalaria was used to compare intervention impact predictions for prevalence and incidence between 2016 and 2020, or 2022.

**Results:**

For each malaria risk stratum four to ten intervention mixes were explored. In the low-risk and urban strata, the scenario without a ITN mass campaign in 2019, predicted high increase in prevalence by 2020 and 2022, while in the very-low strata the target prevalence of less than 1% was maintained at low pre-intervention transmission intensity and high case management. In the moderate and high strata, IPTsc in addition to existing vector control was predicted to reduce the incidence by an additional 15% and prevalence by 22%. In the high-risk strata, all interventions together reached a maximum reduction of 76%, with around 70% of that reduction attributable to high case management and ITNs. Overall, the simulated revised NMSP was predicted to achieve a slightly lower prevalence in 2020 compared to the 2015–2020 NMSP (5.3% vs 6.3%).

**Conclusion:**

Modelling supported the choice of intervention per malaria risk strata by providing impact comparisons of various alternative intervention mixes to address specific questions relevant to the country. The use of a council-calibrated model, that reproduces local malaria trends, represents a useful tool for compiling available evidence into a single analytical platform, that complement other evidence, to aid national programmes with decision-making processes.

**Supplementary Information:**

The online version contains supplementary material available at 10.1186/s12936-022-04099-5.

## Background

Since 2000, increased funding towards the universal scale-up of malaria control prevention mainly through insecticide-treated bed nets (ITNs), and treatment with artemisinin-based combination therapy (ACT), substantially reduced the malaria transmission and burden in sub-Saharan Africa [1, 2]. However, in recent years, progress has stalled, and many countries are not on track to achieving national and global targets for 2020 and 2025 as defined in the World Health Organization (WHO) Global Technical Strategy [3]. Mainland Tanzania and nine other countries in Africa contribute together to 66% of the global malaria burden [4]. In 2018 the persistent high burden has instigated the ‘high burden to high impact’ (HBHI) approach for an improved allocation of limited resources in national malaria strategic plans [4]. This approach, encourages national malaria control programmes (NMCPs) in high malaria burden countries to include sub-national stratification of malaria risk with targeted interventions, thereby allowing to intensify control efforts in high transmission areas, while maintaining the gains achieved in low transmission areas. In this context, a rigorous approach for sub-national tailoring of interventions that consist in appropriately selecting intervention mixes for specific risk areas remains a challenge and requires a good understanding of the local context. This can be informed with the use of mathematical modelling to predict the impact that different strategies might have.

Data from epidemiological, clinical, and operational studies along with routine health information systems represent a valuable data source for informing on malaria trends and retrospective intervention impact. However, they can be limited in predicting the impact of interventions over time in specific geographies and for specific combinations of interventions [5]. Mathematical models represent a powerful tool for simulating setting specific malaria transmission dynamics and quantify, with some level of uncertainty, the impact of interventions and their combination in such settings. Mathematical modelling has been used to inform global and national strategies [6–9], develop target product profiles for new interventions, investigate potential intervention combinations and alternatives (e.g. vector control [10], or vector control and [11]), predict the impact of new interventions such as vaccines [12], and to understand the potential role of surveillance-response [13], among other. The application of mathematical modelling at country level with simulations of suggested intervention mixes for specific geographies has a strong potential to aid decision-making and facilitate a better strategic approach in the selection of interventions.

In mainland Tanzania, the overall malaria prevalence decreased from 18% in 2008 [14] to 7.3% in 2017 [15]. As prevalence declines, the heterogeneity in malaria transmission has increased with 40% of the population living in areas of low or very low malaria risk (prevalence among school children five to sixteen years old *Pf*PR_5-16_ < 1 and < 5%), 23% living in moderate risk areas (*Pf*PR_5-16_ 5–30%), and 37% in high-risk areas (*Pf*PR_5-16_ > 30%) that are predominantly located in the north-west and south-east of the country [16]. In the 2015–2020 National Malaria Strategic Plan (NMSP) intervention packages were defined at the regional (admin 1) level with ITNs distributed through mass campaigns or school net programmes (SNP), indoor residual spraying (IRS) in the Lake Zone and larval source management (LSM) in some urban areas with a national target prevalence of less than 1% by 2020 [17]. In 2017, mathematical modelling was applied to assess technical feasibility of reaching the NMSP target and to determine which intervention mixes would maximize impact to meet the target and within the constraint of cost-effectiveness [18]. In alignment with the modelling results, the mid-term strategic review of the NMSP in 2017, concluded that national prevalence targets would not be achieved with the current strategy [19].

In response to the outcomes of the mid-term strategic review, the Tanzanian NMCP developed an innovative approach to stratify the epidemiological risk of the country at the council (admin 2) level as described in Thawer et al. [16]. In the stratification multiple malaria risk indicators from school survey and routine health surveillance data were combined into an overall malaria risk score per council. The resulting risk map grouped the 184 councils of the mainland into four epidemiological strata: very low (*Pf*PR_5-16_ < 1%), low (*Pf*PR_5-16_ 1–5%), moderate (*Pf*PR_5-16_ 5–30%), and high (*Pf*PR_5-16_ > 30%), and one operational stratum, urban.

In 2018, recommendations from a consultative meeting [21] with a group of national and international malaria experts, selected a series of intervention mixes to be simulated for each strata including interventions not yet implemented in countries and/or for which WHO guidance was lacking (see complete list in Additional file 1).

The selection of appropriate interventions for each of these strata was performed in partnership with mathematical modellers who provided simulated evidence to compare the impact of various pre-determined intervention mixes.

In this process, three questions were of particular interest: (a) What would be the impact of stopping ITN mass distribution campaigns in the very-low, low, and urban strata? Discontinuing mass campaigns in these areas might allow redistribution of resources for intensified efforts in high-risk areas. (b) What would be the additional benefit of intermittent preventive therapy in school-aged children (IPTsc) when ITNs (and/or IRS) are already deployed in moderate and high-risk strata? School children were found to have high malaria prevalence with high contribution to the transmission in the community [22], and preventive therapy in children might be a valuable addition to the intervention mix deployed in countries [23]. (c) What combination of interventions would be required to substantially reduce malaria in the high risk-stratum? Despite the successes in the past, malaria transmission is still intolerably high in many parts of the country, mainly rural areas [24]. This question aimed to assess the hypothetical maximum impact with the interventions considered for the new strategic plan that could be reachable if funding was not limited. In addition to these questions, it was of interest to compare the predicted impact of interventions defined in the 2015–2020 NMSP with a new candidate strategy for a supplementary strategic plan for the years 2018–2020.

This paper presents the modelling approach conducted in 2018, based on a previous model calibrated to the councils until 2016, used to support answering these specific programmatic questions. The results of the present analysis provided additional information for the NMCP to update their national strategic plan for the period 2018–2020 [20].

## Methods

### Update of the National Malaria Strategic Plan

**Fig. 1:**
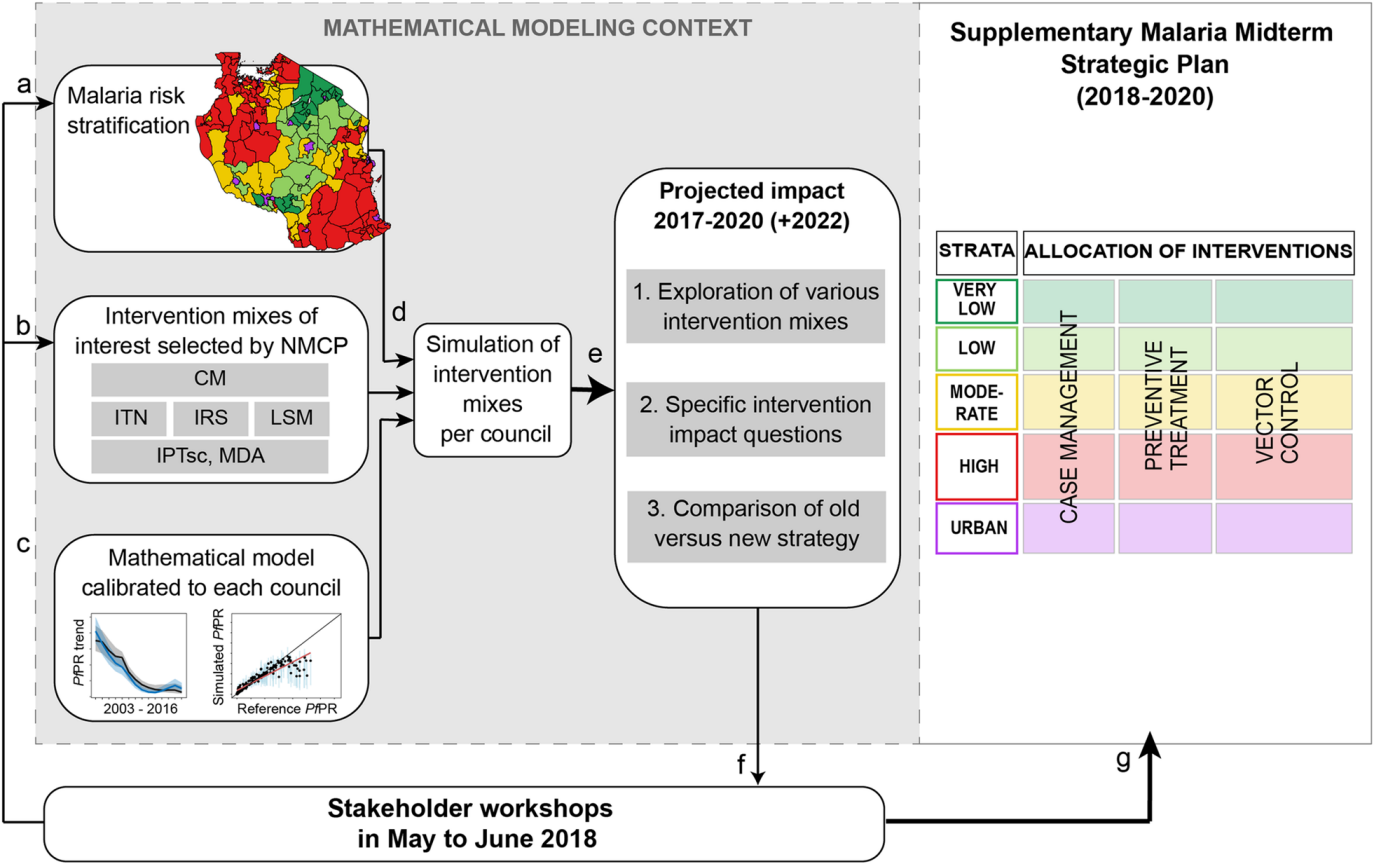
Overview of modelling support in the process of sub-national tailoring of malaria interventions in Mainland Tanzania. Initial strategic planning meeting [21] that followed the expert consultative and midterm strategic planning meeting (see Runge et al. 2020 [25] for full timeline), that led to the **a** malaria risk stratification [16] as well as **b** pre-selection of potential intervention mixes per strata. **d** The malaria risk stratification and the intervention mixes together with a previously calibrated malaria transmission model [18] were used to generate intervention impact predictions per council. **e** The objective for the simulations and respective results were 1. the exploration of various intervention mixes at target coverage levels with or without strengthened case management, 2. the comparison of specific intervention scenarios to address some of the questions most relevant to the NMCP at the time of the analysis (questions denoted as **a**–**c** in results), and 3. the comparison of the simulated current strategy against the potential new strategy. **f** Modelling results together with NMCP performed analysis and review were discussed. **g** Formulation of the supplementary malaria midterm strategic plan (2018–2020 NMSP) [20]

**Fig. 2:**
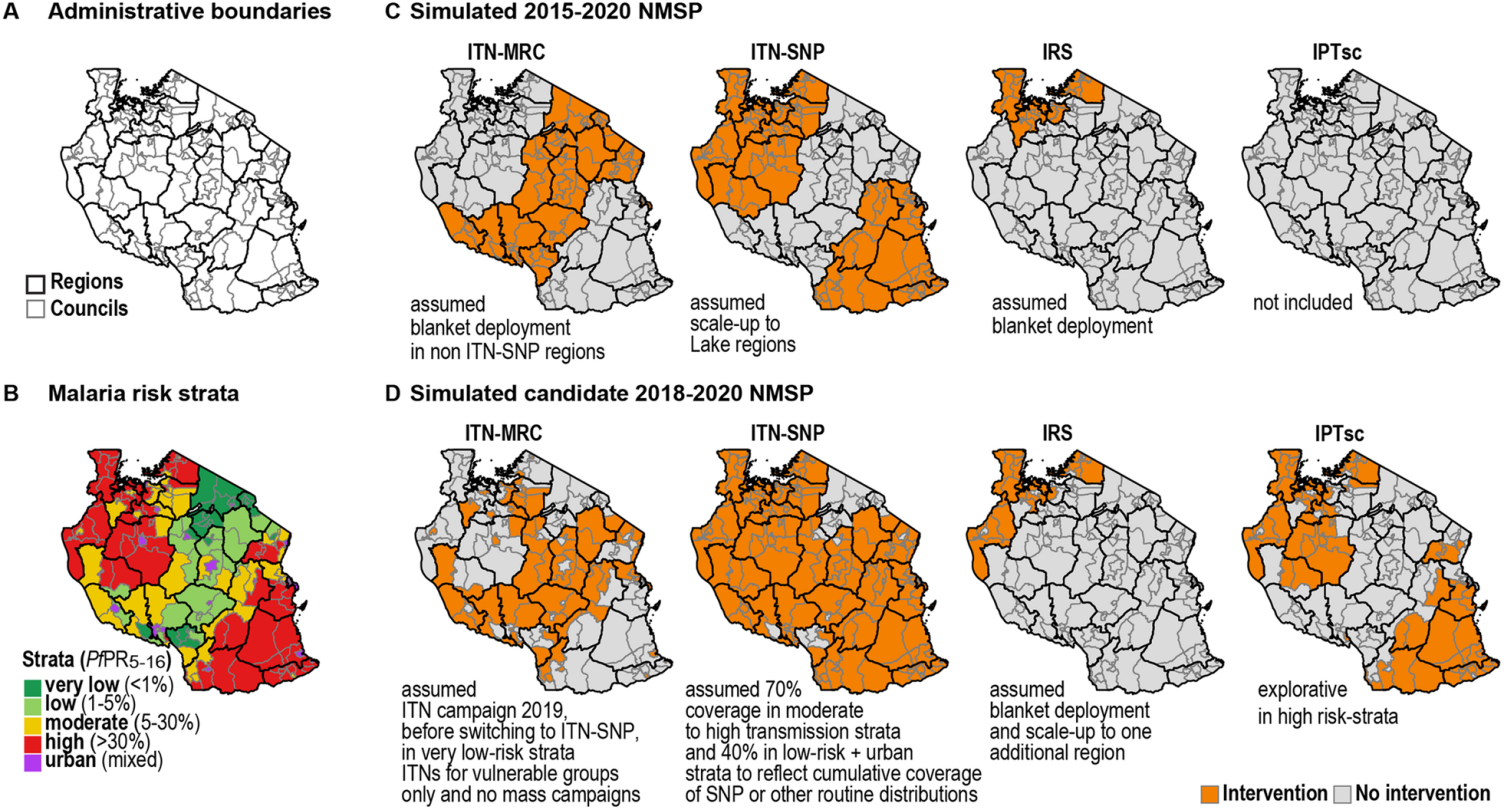
Overview of **A** administrative boundaries, **B** malaria risk stratification and **C**, **D** simulated interventions corresponding to simplified versions of the 2015–2020 and 2018–2020 NMSP per council in Mainland Tanzania. Continuous ITN distribution through schools (ITN-SNP) was operational in fourteen regions (Katavi, Kagera, Mara, Mwanza, Simiyu, Shinyanga, Geita, Lindi, Mtwara, Ruvuma, Morogoro, Tabora, Kigoma, Pwani), and in those areas no ITN-MRC was planned for 2019. ITN-SNP was simulated with 70% coverage in moderate to high transmission strata to reflect school net distributions, or with 40% in the low-malaria risk strata and in urban councils to reflect cumulative coverage of other routine distributions schemes. The final intervention allocation can be obtained from the supplementary malaria midterm strategic plan (2018–2020 NMSP) [20]. ITN, Insecticide Treated Nets; IPTsc, Intermittent preventive therapy in school children; IRS, Indoor Residual Spraying, MRC, Mass Replacement Campaign; SNP, School Net Programme

Following the mid-term strategic review in 2017, the NMCP decided to update their NMSP and introduce a new malaria risk stratification in mainland Tanzania [16, 20]. The selection of appropriate interventions in each stratum was discussed in consultative meetings held in 2018 [21] with various stakeholders including local researchers, funders, implementers, interregional collaborators, and international partners. A ‘Strength, Weakness, Opportunity and Threat’ (SWOT) analysis was conducted by focus area (integrated malaria vector control, case management, surveillance and monitoring, social behaviour and change communication, and program management), to help in the selection of potential intervention packages for each of the strata [21]. Mathematical modelling was then used to simulate the impact of these suggested intervention mixes per council in order to support the revision of the strategic plan. The revised strategic plan was released in the Supplementary Malaria Midterm Strategic Plan 2018–2020 [20], in the following referred to ‘2018–2020 NMSP’. An overview of the process and methods in shown in Fig. 1 and comparison of the simulated interventions in previous and revised NMSP in Fig. 2.

### Model parameterization and calibration

The microsimulation platform OpenMalaria [26] was used to simulate the dynamics of malaria and the impact of interventions in each council. This malaria transmission model represents the dynamics of malaria in humans with an individual-based model and includes a population model simulating the vector dynamics [27–34] as well as the effect size of interventions [10, 29, 35–37]. The within-host component of the model had been previously calibrated to historical studies [27, 31]. Transmission seasonality and intensity, historical intervention coverage and vector bionomics are key parameters that characterize a council and were informed by country specific data. Data sources included national-level household surveys and malaria indicator surveys, as well as entomological surveillance reports and intervention distribution information available from the NMCP. Details about the model parameterization and calibration have been described elsewhere [18]. In brief, parameter estimates for historical ITN usage for 2003–2010 were obtained from the Malaria Atlas Project (MAP) [38] and for 2010, 2012 and 2016 from the national-level household survey data [39–41]. Model assumptions on treatment seeking behaviour were informed by estimates from the national-level household survey data 2016 combined with a country specific scaling factor for effective treatment given access to health services from Galactionova et al*.* [42]. Geospatial predictions of *Plasmodium falciparum* prevalence among children aged 2 to 10 years (*Pf*PR_2to10_) were derived for each council between 1990 and 2017, based on various community surveys conducted in Tanzania [2]. Using a Bayesian framework, the model was fitted to these predictions to represent historic trends of malaria transmission in each council [18]. Estimated parameters during the fitting process were the pre-intervention annual entomological inoculation rate (EIR) in 2003, and the ITN decay of nets distributed in 2012 for councils that had a mass campaign, or the ITN coverage between 2012 and 2016 for councils with annual school net distributions [43]. Population estimates were obtained from the national census in 2012, assuming linear growth between 2012 and 2016, and constant population afterwards [44]. A constant importation rate of 5 cases per 1000 population per year was used, corresponding to the estimated range of importations with Zanzibar [45]. The model was calibrated for each council and fitted to the model-based prevalence estimates per year between 2003 and 2016. The total simulation time spanned from 2003 to 2022, capturing historical trends until 2016 and projected impact of intervention combinations of interest from 2017 to 2020, with 2 additional years for follow up. In the model, no explicit distinction between urban and rural councils was made. Details on fitting performance per strata are provided in Additional file 2.

### Characteristics of simulated interventions 2017–2020

Seven different interventions were simulated either alone or in combinations. The intervention coverage, efficacy and deployments are summarized in Table 1. In the simulations, “effective treatment rates” also referred to as “case management” (CM) coverage were defined as the proportion of uncomplicated malaria cases effectively cured after treatment with an anti-malarial using a parameterization corresponding to ACT, with 98% efficacy in clearing blood stage parasites. As for historical case management, the effective coverage parameter indirectly took into account treatment-seeking and treatment failure rates [42]. The effective treatment coverage for severe malaria cases was fixed at 48% [46].

**Table 1:** Simulated interventions, deployment, coverage, and efficacy

	Deployment		Efficacy		
	Coverage	Timing	Active ingredient/Efficacy mechanism	Resistance	Duration
CM	As in MIS 2016 [41] or 85%	Jan 2017	ACT	0%	Constant
ITN-MRC	80%	Jan 2019	pyrethroid	80%	50% effectively used after 3 years
ITN-SNP	40%, 70%	Jan 2017–2020	pyrethroid resistance	80%	50% effectively used after 3 years
IRS	85%	Sep 2017–2020*	Actellic 50EC, Bendiocarb	0%	3 months (Actellic 50EC) 6 months (Bendiocarb)
LSM	60%	Setting specific at peak in transmission 2017–2020	Reduction of emerging adult mosquitoes	–	4 months of constant effectiveness
IPTsc	80% 5–16 years	Jun, Oct 2017–2020	Immediate clearance of blood-stage parasites	–	2 weeks prophylactic effect
MDA	80%	Jun, Aug, Oct 2017–2020	Immediate clearance of blood-stage parasites	–	No prophylactic effect

* Applying in rotation Bendiocarb and Actellic 50EC, starting with Bendiocarb in 2017

Coverage of ITNs was defined as the ratio of the population protected by bed nets amongst the total population at risk. The ITN distributions were simulated so that (a) coverage levels (proportion of population protected by a net) increased either at once, representing mass replacement campaigns (ITN-MRC), or (b) coverage were maintained at constant levels, representing continuous distribution mechanisms [43]. In Tanzania, these continuous delivery mechanisms include school net programmes (SNP) and distribution through reproductive and child health clinics (RCH). In the model, the effect of both continuous ITN distribution mechanisms were simulated with a higher and lower cumulative coverage level and both abbreviated using ITN-SNP for simplicity. The ITNs were assumed to have a half-life of 3 years, inferring that only 50% of these would still be used after three years [47] and for conservative purposes, it was assumed that high insecticide resistance to pyrethroids was established throughout the country for all vectors.

The simulations for IRS assumed the insecticide had organophosphate (Actellic 50EC) or carbamate (Bendiocarb) as active ingredients, as these were the used insecticides in recent years [48]. The effect size parameterization was already established previously, with Actellic 50EC parameterized based on an experimental hut study In Côte d’Ivoire [49] and Bendiocarb based on a study in Benin [50] (Briët et al., pers. commun.). Annual rotation between these insecticides was assumed starting with Bendiocarb in September 2017.

Although mass drug administration (MDA) had not been implemented in the country, the intervention was explored in the simulations, assuming an immediate clearance of blood-stage parasites without any lasting prophylactic effect after administration and targeting the whole population.

LSM, specifically with larviciding, was simulated with an effective coverage defined as the proportion of larvae killed (when compared to the number that should have emerged).

For intermittent preventive treatment in school-aged children (IPTsc), targeting children aged 5 to 16 years it was assumed that the drug would lead to immediate parasite clearance and have a prophylactic effect of fourteen days. Seasonal malaria chemoprevention (SMC) was considered for only six eligible councils was not specifically simulated, although roughly approximated with IPTsc in the strategic plan simulations. Intermittent preventative treatment in pregnant women (IPTp) is included in the national strategy but not in the simulations as its impact on transmission is limited [35]. Similarly, behaviour change communication was not explicitly modelled as its direct impact is difficult to quantify. For all simulated interventions it was assumed that the coverage and the effectiveness would be homogeneous within each council.

### Simulated intervention mixes and national malaria strategic plans

For the explorative analysis, full factorial combinations of the interventions described above were simulated for all councils. The resulting dataset included model predicted annual outcomes per council per intervention combination for 2016 (baseline) to 2020 (comparison), and 2022. To form a specific intervention mix for the country, the dataset was filtered to match selected intervention mixes to the respective councils. Hence, the simulated national malaria strategic plans were simplified versions, excluding IPTp and IPTi as well as novel insecticides, such as Piperonyl butoxide treated nets (2 councils) or SumiShield. SMC, planned in six councils, was approximated with IPTsc. IRS or LSM did not distinguish between targeted or blanket deployment. Strengthened CM was simulated with a target coverage of 85% effective treatment. The country stratification and simulated interventions are shown in Fig. 2.

### Analysis of simulation results

The model outputs were summarized with prevalence rates among children aged between two and ten years and incidence in the whole population, defined as the total number of new cases (i.e. uncomplicated and severe malaria episodes) within a year per 1000 population. Model estimates for council predictions are summarized with median and credible intervals from the posterior distribution from the model calibration. In addition, estimates per strata and at national level were summarized with population-weighted means and 95% confidence intervals (95%CI, shown in squared brackets) of the council median estimates.

Relative reduction between 2016 and 2020 was calculated as (x_2016_-x_2020_/ x_2016_) *100, with x being either prevalence or incidence per council or strata. The impact of discontinuation of ITNs mass campaigns (very low-risk, low-risk, and urban strata) was described by comparing predicted prevalence and percent point change in prevalence between the scenario with strengthened CM only (discontinued vector control interventions starting in 2017) and the scenario with ITNs deployed as a mass campaign (ITN-MRC in 2019) aggregated per strata or per council and pre-intervention EIR within the very low risk strata.

The impact of IPTsc (moderate and high risk-strata) was estimated by calculating the relative reduction in prevalence and incidence compared to no IPTsc, using predictions for 2020. The impact of strengthened CM was assessed in the same way using current CM levels as the counterfactual scenario.

The incremental benefit of adding interventions in the high-risk strata was described using the relative reduction for each intervention mix between 2016 and 2020. The intervention scenarios were: (1) no intervention other than strengthened CM; (2) ITN-MRC; (3) ITN-MRC in combination with IPTsc; (4) ITN-MRC in combination with IRS; (5) ITN-MRC in combination with ITN-SNP; (6) ITN-MRC in combination with ITN-SNP and IPTsc; (7) ITN-MRC in combination with ITN-SNP and IRS; and (8) ITN-MRC in combination with ITN-SNP, IRS and IPTsc. All eight scenarios were simulated assuming strengthened CM.

To compare the impact of the intervention mixes of the two NMSP’s, the difference in predictions was computed for each council (X_2015-2020_NMSP_—X_2018-2020_NMSP_, with X being either prevalence or incidence in 2020). The council predictions were aggregated per strata (and nationally) using unweighted and population weighted means for comparison. All analyses were performed using R and RStudio (R Core Team, 2020).

## Results

### Impact of strata-specific intervention combinations for 2017–2020

**Fig. 3:**
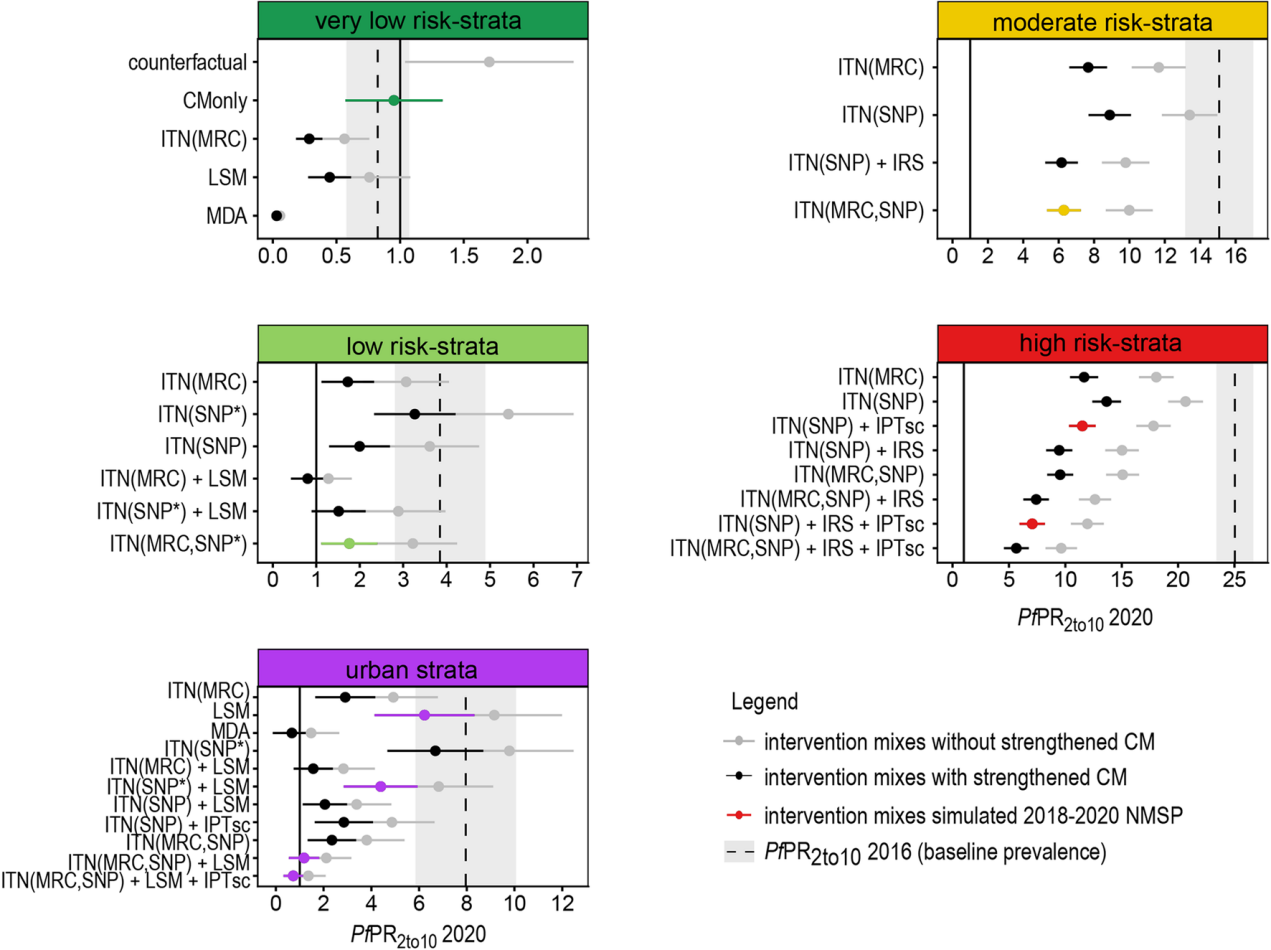
Predicted prevalence for intervention mixes per strata for 2020. The error bars and shaded area show the mean and 95% confidence intervals based on heterogeneity among councils. The black and highlighted error bars correspond to current CM level simulations and the grey error bars to strengthened CM. The highlighted error bars show the intervention combinations selected for the 2018–2020 NMSP. The vertical solid line indicates a prevalence of 1% and the dashed line shows the simulated prevalence for 2016

Four to eleven scenarios with different intervention mixes and two levels of CM were simulated for an explorative comparison per strata, with fewest scenarios in the moderate risk and most scenarios in the urban stratum (Fig. 3).

In the *very low-risk stratum*, the predicted mean prevalence for 2020 ranged from < 0.1% to 1.5% across the simulated interventions and for both CM levels. Maintaining current CM level without additional interventions (counterfactual) was the least effective and implementing MDA was found to be the most effective intervention. Strengthening of case management, the intervention scenario selected for the 2018–2020 NMSP showed a predicted prevalence in 2020 close to the baseline prevalence in 2016.

In the *low-risk stratum*, the predicted prevalence for 2020 ranged from 0.7% to 4.7% across the simulated interventions. In this stratum, the coverage of ITNs and delivery mechanisms were drivers of impact. Prevalence in 2020 had comparable low levels either when mass campaigns were implemented or if the coverage was at least of 70% when distributed continuously. Amongst the suggested strategies, the ITN mass campaign combined with implementation of LSM had the most impact with predicted prevalence of 0.7% [95% CI 0.5–0.9%] in 2020. On the contrary, a continuous distribution of nets with low coverage (40%) showed the lowest impact, especially with low CM levels (4.6% [95% CI 4.0–5.4%]). All scenarios had on average a lower prevalence compared to the 2016 baseline level (*Pf*PR_2to10_2016: 3.8% [95% CI 2.9–4.9%]), except for ITN continuous distribution with 40% coverage and current CM.

In the *moderate-risk stratum*, all four simulated scenarios resulted in lower prevalence in 2020 than in 2016 (*Pf*PR_2to10_2016: 15.3% [95% CI 13.2–17.3%]), that was further reduced when assuming strengthened CM, with prevalence ranging from 5.0% to 10.8%. The implementation of both ITN delivery mechanisms simultaneously, as included in the 2018–2020 NMSP, and assuming 70% coverage, was predicted to have similar impact as ITN school campaigns coupled with the implementation of IRS (5.1%, [95% CI 4.6–5.6%] vs 5.0% [95% CI 4.5–5.5%]).

In the *high-risk stratum*, the predicted prevalence for 2020 ranged between 4.5% and 16.2% across the suggested scenarios, all lower than the baseline prevalence of 25.2% [95% CI 23.6–26.7%]. The simulations showed that the implementation of both ITN delivery mechanisms simultaneously demonstrated a lower prevalence compared to either one alone or in combination with other interventions (7.5% [95% CI 7.0–8.1%] for ITN-MRC + ITN-SNP; 9.2% [95% CI 8.5–9.8%] for ITN-MRC; 10.7% [95% CI 10.1–11.5%] for ITN-SNP). The most impactful intervention mix corresponded, as expected, to the scenario with the most interventions (both ITN distribution mechanism, IPTsc and IRS) and was expected to lower the prevalence to 4.5% [95% CI 4.0–5.0%] in 2020. The intervention mixes simulated for the 2018–2020 NMSP were ITN-SNP, IPTsc and IRS in some councils, the associated predicted prevalence for 2020 was 5.6% [95% CI 5.2–6.2%] with IRS and 9.0% [95% CI 8.4–9.7%] without IRS.

The *urban stratum* included a broad mix of intervention scenarios, ranging from single interventions such as strengthened CM, LSM, or MDA to combinations of those in addition to either ITN mass or continuous campaigns, depending on the epidemiological strata. The predicted prevalence of the simulated interventions ranged between 0.6% and 7.7%. All intervention combinations were predicted to reduce the prevalence compared to 2016 (*Pf*PR_2to10_2016: 8.1% [95% CI 5.9–10.2%]), except LSM with current CM. In this stratum, four intervention combinations were suggested for the 2018–2020 NMSP depending on the epidemiological risk-strata.

### Impact of discontinuation of ITN mass campaigns in very low, low, and urban strata

**Fig. 4:**
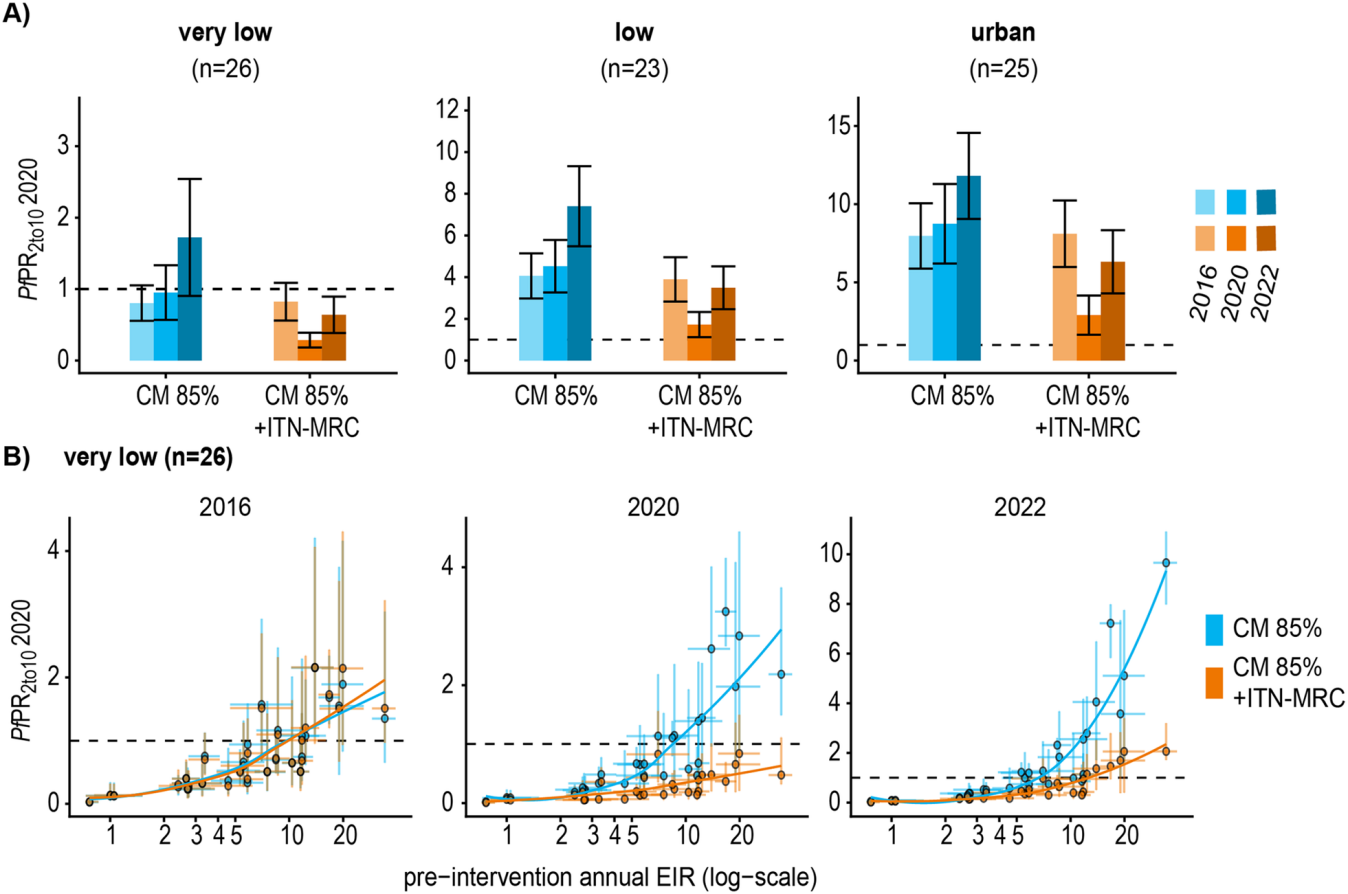
Predicted impact on prevalence for discontinuation of ITN mass campaigns in the very low, low, and urban strata. **A** The bar charts show the mean and 95% confidence interval of council prevalence aggregated per strata comparing improved CM without ITN-MRC distribution in 2019 (blue) to improved CM with ITN-MRC distribution in 2019 (orange). **B** Prevalence per pre-intervention transmission intensity per council in the very low-risk stratum compared for the two scenarios with or without additional ITNs for the years 2016, 2020 and 2022. The points indicate the posterior median, the error bars and the credible intervals resulting from the model calibration. The dashed line highlights a prevalence of one percent

Without an ITN mass campaign in 2019, an increase in prevalence was predicted in all the three strata with moderate increase by 2020 and considerable increase by 2022. For 2020, the absolute increase was marginal in the very low-risk strata (0.53 [95% CI 0.37–0.70] percent points), slightly higher in the low-risk strata (2.17 [95% CI 1.71–2.62] percent points), and around 5 percent points in the urban strata (5.2 [95% CI 4.3–6.1] percent points) (Fig. 4A). The magnitude in increase differed depending on the pre-intervention transmission intensity. Among the councils in the very low-risk strata, the prevalence remained below the 1% threshold until at least 2022 at pre-intervention EIRs lower than 7 infectious bites per person per annum (ibpa). At pre-intervention EIRs lower than 10 ibpa, the prevalence remained below 1% by 2020, and at pre-intervention transmission levels above 10 ibpa, a prevalence below 1% could not be maintained or reached in 2020 and increased further by 2022 (Fig. 4B).

### Potential benefit of adding IPTsc in the high and moderate-risk strata combined

**Fig. 5:**
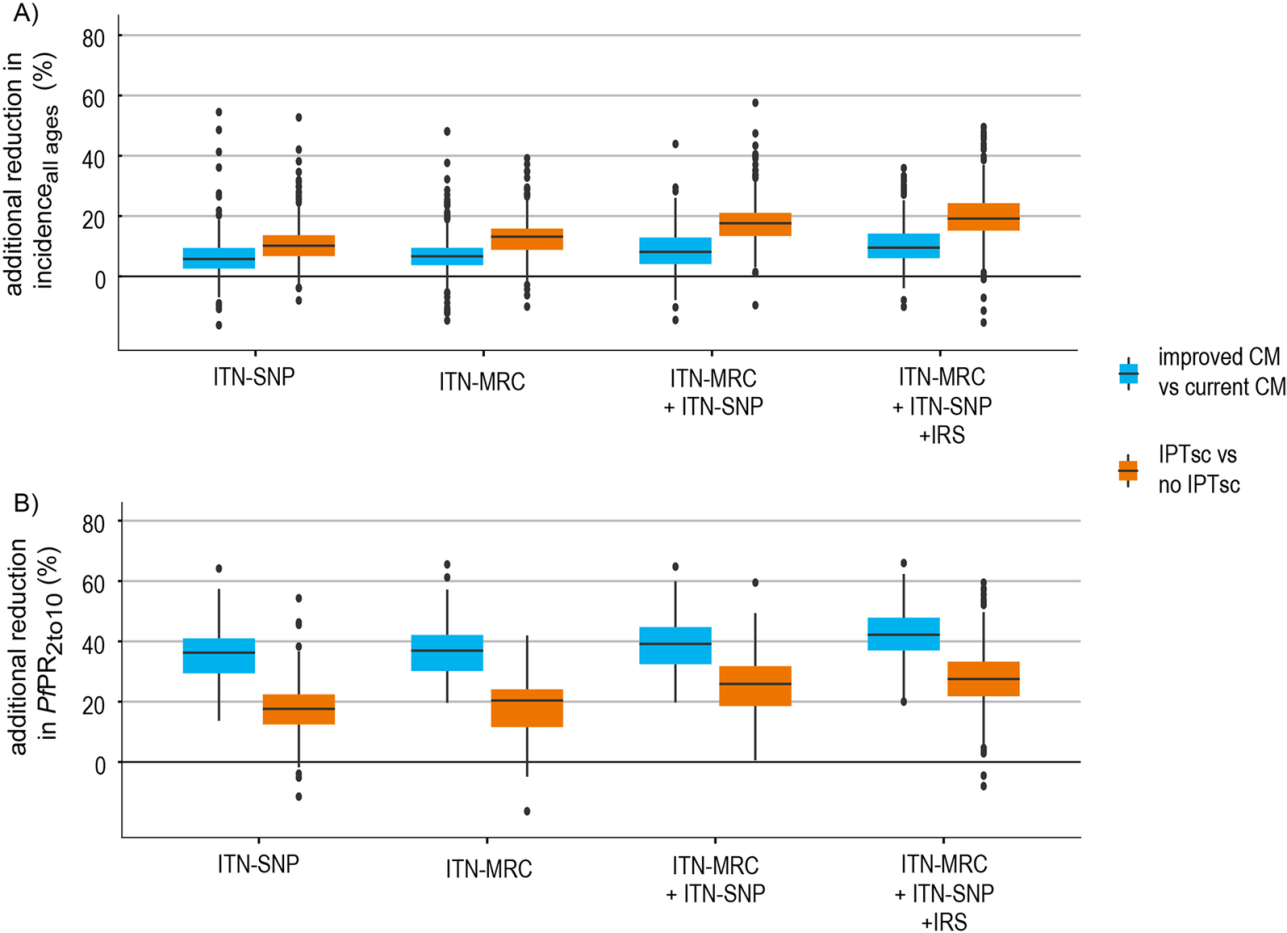
Predicted impact of IPTsc and CM in the moderate and high-risk strata combined. **A** The impact on incidence in the total population for 2020 and **B** on *Pf*PR_2to10_ for 2020. The impact of IPTsc was evaluated when deployed on top of vector control interventions and the impact of strengthened CM was plotted as reference. The boxplots show the distribution among councils in the moderate- and high-risk strata combined. The x-axis presents the different combinations of vector control interventions

IPTsc reduced the predicted prevalence for 2020 on average by 22.3% [95% CI 21.8–22.8%] and the incidence by 15.2% [95% CI 14.7–15.6%], when combined with vector control intervention mixes (ITN-MRC, ITN-SNP, IRS). In comparison to IPTsc, strengthening CM was less effective in reducing incidence in all ages (8.2%, [95% CI 7.8–8.5%], while more effective in reducing prevalence in children between 2 and 10 years (38.9%, [95% CI 38.6–39.4%]) (Fig. 5).

### Incremental benefit of interventions in the high-risk stratum

**Fig. 6:**
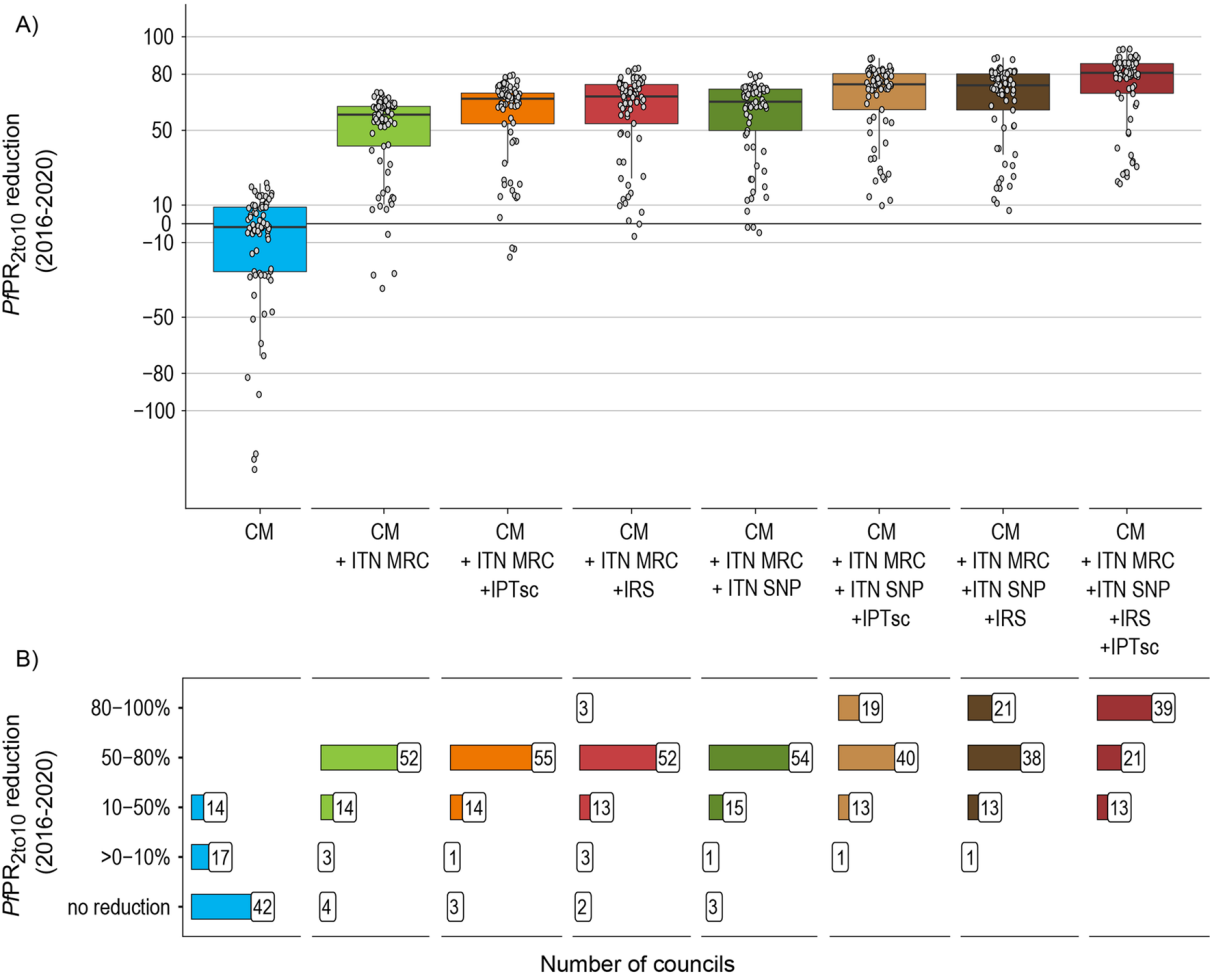
Predicted reduction in prevalence in 2020 compared to 2016 per intervention combinations for councils in malaria high-risk strata. **A** Predicted prevalence reduction between 2016 and 2020 per incremental intervention mix, each point represents a council (n = 73, including four urban councils). The solid line in the boxplot shows the median and the dashed line the mean. **B** Number of councils per incremental intervention mix grouped by prevalence reduction

The choice of the intervention mixes considered in the high-risk stratum were made to understand the incrementable benefit of each additional interventions. The simulated scenario that corresponded to a maximum number of interventions (both ITN delivery mechanism, strengthened CM, IRS and IPTsc) predicted an average reduction in prevalence by 76.6% [95% CI 72.6–80.7%] between 2016 and 2020. Of that reduction, approximately 70% was attributed to strengthened CM combined with ITN-MRC, which was predicted to reduce the prevalence on average by 52.0%, [95% CI 47.1–57.9%] (Fig. 6A). The other intervention mixes, as shown in Fig. 6A, were predicted to reduce the prevalence by an additional 8% to 25%. The relative reduction in prevalence of additional IRS was comparable to that of additional IPTsc or ITN-SNP (relative reduction in *Pf*PR_2to10_ 63.1% [95%CI 58.4–67.9%] for IRS, 60.9% [95%CI 56.3–65.5%] for IPTsc, 61.1% [95%CI 56.7–65.4%] for ITN-SNP when administered in addition to strengthened CM and ITN-MRC) (Fig. 6A). Strengthened CM and ITN-MRC were predicted to reduce the prevalence by at least 50% in most councils (n = 52 out of 73 districts in high strata including urban high-risk councils). However, in at least 13 councils, even the implementation of all interventions would not be sufficient to reduce prevalence by more than 50% (Fig. 6B). The 13 councils were located in the Southern Zone for which estimated pre-intervention EIR’s were highest, and prevalence predictions started to increase before 2020 (Additional file 2, Fig. A2.5).

### Predicted impact of the simulated 2015–2020 NMSP and 2018–2020 NMSP

**Fig. 7:**
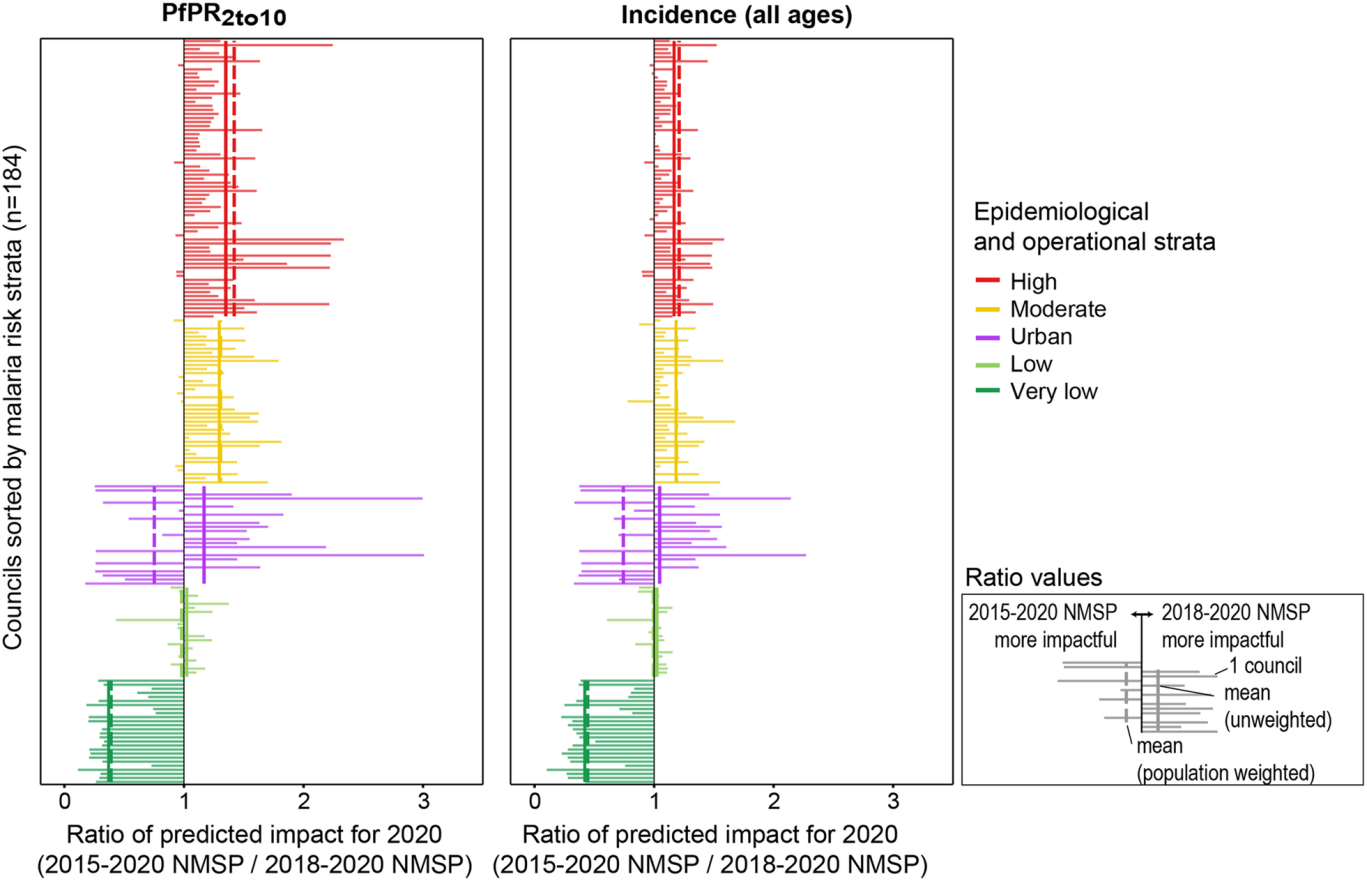
Comparison of predicted impact of the simulated 2015–2020 NMSP and 2018–2020 NMSP per council for 2020. Ratio in predicted prevalence (left panel) and incidence (right panel) for 2020 between both simulated NMSPs. Each horizontal line represents one council. The vertical lines show the mean ratio per strata with solid line for unweighted mean and dot-dashed line for population-weighted mean

In the simulation of the 2018–2020 NMSP, the prevalence reductions between 2016 and 2020 were highest in the high and moderate-risk strata (mean *Pf*PR_2to10_ reduction 63.5%, [95% CI 57.8–69.2%] and 58.9%, [95% CI 55.7–62.1%], respectively), followed by the low- and urban risk strata (57.8%, [95% CI 53.4–62.3%] and 60.1%, [95% CI 52.8–67.3%], respectively) and the lowest in the very low-risk strata (1.5%, [95% CI -12.7–15.8%]) (Additional file 2, Fig A2.4, Table A2.1). Figure 7 shows the ratio of the predicted prevalence and incidence for 2020 of both simulated NMSPs. Compared to the simulated 2015–2020 NMSP scenario, the simulated 2018–2020 NMSP scenario projected lower prevalence and incidence values for most of the councils in the moderate and high-risk strata (with mean ratio of for both strata respectively). In the low-risk strata the simulated 2015–2020 NMSP performed better, while the impact was heterogeneous in the low-risk and in the urban strata. The mean across councils was the same or very similar to the population weighted mean across councils in all strata, except in the urban strata (Fig. 7). In the simulation of the 2018–2020 NMSP, the proportion of the population that would live at a high malaria risk (*Pf*PR_2to10_ greater than 10%) in 2020 would be around 10% less than in the simulation of the 2015–2020 NMSP (Additional file 2, Table A2.2).

## Discussion

In this work, mathematical modelling was used to simulate a set of selected intervention mixes between 2017 and 2020 (+ 2022), tailored to the malaria risk strata at council level in mainland Tanzania. The simulated strata-specific intervention mixes were selected to (i) represent the 2015–2020 [17] and the 2018–2020 NMSP [20]; (ii) and to address specific questions relevant to the NMCP. These questions were: (a) what would be the impact of stopping ITN mass distribution campaigns in the very-low, low, and urban strata; (b) what would be the additional benefit of IPTsc when ITNs (and/or IRS) are already deployed in moderate and high-risk strata; and (c) what intervention mix would be required to substantially reduce malaria in the high risk-stratum. The simulations and main analyses were conducted during the strategic planning process in 2018 [25] that accompanied the development of the council-level malaria risk stratification [16]. This work utilizes a previously parameterized model, calibrated to each council in mainland Tanzania, to provide timely model predictions interactively discussed with the NMCP. A table for each of the simulated intervention scenario per strata and the corresponding strategic response was included in the 2018–2020 NMSP [20] (Additional file 3). The process and lessons learned have been published elsewhere [25] and the discussion below focuses on the technical aspects of the modelling analysis.

Overall, both simulated NMSPs had similar impact predictions at national level, that highly varied among and within risk strata. For instance, in the high- and moderate-risk strata the 2018–2020 NMSP was predicted to achieve higher reductions than the 2015–2020 NMSP, which in turn performed slightly better in the very low-risk stratum. Impact predictions for the low-risk and urban strata were mixed and did not show a clear tendency for either strategy. Although, substantial reductions in prevalence for all strategies were predicted at national level between 2017 and 2020, the reductions were not enough to reach the 2015–2020 NMSP target prevalence of less than 1% by 2020. Notably, in both simulated NMSPs the impact on prevalence, with strengthened CM in addition to vector control, was higher than the impact gained through the reallocation of vector control or additional IPTsc for the time frame in consideration. Such a high effect from strengthened CM in combination with vector control was also observed in other countries [8, 51].

In mainland Tanzania, most of the councils in the very low-risk stratum have low receptivity due to unsuitable climate and environmental conditions [52], hence a sub-analysis of the simulations assessed the scenario of discontinuing large scale ITN mass campaigns in the very-low and low risk strata as well as urban strata. The results showed clear increasing trends by 2020 in the low-risk and urban strata for the scenario without an ITN-MRC in 2019 and an even higher increase by 2022, whereas predictions were not homogeneous among councils. In the very low-risk strata, the increase by 2020 was on average low but highly varied depending on the pre-intervention EIR. At low pre-intervention EIR, the prevalence predicted for 2020 was not substantially higher than with a ITN mass campaign in 2019. However, at higher pre-intervention EIR, high CM alone was not enough to keep prevalence low, which aligns with previous modelling studies [53, 54]. While the results could suggest that universal coverage and large-scale deployment of nets might not be required to maintain baseline levels in low-risk settings with persistent low transmission, the results should be interpreted with caution as local contextual factors, especially surveillance and monitoring capacities, were not captured in the model, and as model stochasticity is usually higher in low transmission simulations.

So far, only very few areas in malaria-endemic countries discontinued ITNs. For instance, in Kenya, ITNs that were previously deployed countrywide [55], are deployed in only 50% of counties in the low-risk zone via routine ITN distributions [56]. Zambia is moving from universal ITN to more targeted campaigns [57], whilst in Namibia, ITNs are mostly recommended for personal protection or in transmission foci in malaria risk free or low transmission areas [58], and in Zimbabwe nets are not distributed in the highland areas that are at very low risk of malaria [59]. Analysis on cancellation of ITN mass campaign due to the COVID-19 pandemic in 2020, in low as well as high transmission areas across Sub-Saharan Africa predicted potential substantial increase in cases and deaths, varying across countries [60]. In practice discontinuation of interventions in persistently low transmission areas should only be considered when having a strong surveillance system in place [53], as well as strategies for foci detection and protection of the vulnerable population as included in the 2018–2020 NMSP [20].

In mainland Tanzania as well as other countries, school children were found to be a large reservoir for malaria parasites [61–63], with malaria prevalence as high as 76% in some councils [61]. A randomized control trial conducted in Ugandan schoolchildren demonstrated high efficacy of preventive therapies in these groups as well as community-wide effect [64, 65]. Results of a recent systematic review showed beneficial impact of preventive treatment in low and high transmission areas [23] and highlights the importance targeting school children in malaria control. IPTsc, simulated in the moderate and high-risk strata, showed an additional benefit on prevalence and incidence independent of the underlying vector control interventions. These results are lower than estimated effectiveness between 66 to 83% combined across field studies [23]. However, a direct comparison is not possible as the in the present analysis IPTsc was simulated on top of strong vector control interventions, and the effectiveness was calculated compared to counterfactual after three years of deployment. Interestingly, IPTsc showed a higher impact on incidence (all ages) but lower impact on prevalence when compared to the impact of strengthened CM. This could be explained by the differences in target population between the two strategies. Simulated IPTsc targets school children aged 5 to 16-years-old, one of the most vulnerable groups that gets highly infected and symptomatic during the transmission season, whereas CM affects the whole population that might have built disease immunity despite high prevalence. Hence, effectively treating infections might reduce transmission in children and adults but affect the burden, especially for the whole population, less. A simplified IPTsc parameterization was used assuming immediate parasite clearance and prophylactic effect of two weeks with four deployment rounds during the transmission season.

In practice, the post-treatment prophylactic effect was found to range between 14 to 35 days and the deployment schedule differed across trials [23], whereas the optimal deployment of IPTsc across different settings is unclear [66]. In practice, concerns also exist about development of resistance in the parasite against the anti-malarials used for IPTsc [67], and further in-depth analysis with varying anti-malarials and deployment schemes would be useful for more accurate IPTsc impact predictions.

In the third sub-analysis of the presented work, it was assessed what it would take to substantially reduce malaria in the high-risk strata by comparing the incremental impact of additional interventions. In the analysis, a high CM coverage was assumed since strengthening the health care system is a priority independent from vector control and other malaria interventions. The model predictions showed a reduction of around 50% for high coverage levels in CM and ITN-MRCs, and a maximum reduction of around 70% when ITN-SNP, IPTsc and IRS were added. In some councils however, additional interventions of one or more of ITN-SNP, IRS, or IPTisc would be needed to achieve at least a 50% reduction in prevalence. While these findings with greatest impact for CM and ITN align with expectations based on previous published studies [38], the high impact predicted for ITNs is to some extent surprising, as high pyrethroid resistance was assumed for all vectors in all areas. This could be due to the high coverage of at least 80% immediately after the mass campaign simulated in 2019 and the continued protection provided by the physical net barrier [68, 69]. The evidence on reduced net effectiveness at different resistance intensities is limited [70] and varies between modelling studies [29, 71]. In addition, the simulated IRS deployments in 2018 and 2020 used the more short-lived Actellic 50EC [72, 73] instead of Actellic 300CS [74, 75], which likely underestimates predicted impact of IRS. An important additional in-depths analysis in mainland Tanzania would be using vector and location specific resistance parameters and including new insecticides for ITNs, such as pyrethroid piperonyl butoxide [76] and IRS (i.e. SumiShield [77]). Moreover, future reassessment of intervention mixes will need to include improved chemoprevention interventions as well as the malaria vaccine, recently endorsed by the WHO [78].

Utilizing a previously calibrated model to address questions that are relevant to the current situation in the country, enabled delivering timely results to meet the needs of the NMCP during the funding application and strategic planning cycle. In this process, the model results provided an additional layer of information for the selection process of targeted interventions per malaria risk strata. While an impactful approach, the modelling analysis has several limitations that affect the accuracy of the results.

First, the simulated NMSPs are a simplified version of the actual NMSPs [17, 20], as not all interventions were simulated. For instance, IPTp and IPTi were excluded as they were assumed not to have a major impact on malaria transmission in the community [35]. Piperonyl butoxide treated nets were not simulated as, at the time of analysis, planned in only two councils [79]. Similarly, SMC, planned in six councils, was approximated with IPTsc. In the model larviciding did not distinguish between targeted or blanket deployment and due to large uncertainties around feasible larviciding coverages and related effectiveness [80], simulation outputs were excluded from results presented to the NMCP. Strengthened CM was simulated with a target coverage of 85% effective treatment, whereas health system strengthening will likely differ across councils as they depend on baseline performance and strategies to improve CM in respect to the local contexts.

Second, the specific questions addressed would benefit from further in-depth analyses at a more granular level and including more sources of uncertainty, i.e. uncertainty in intervention parameters and model structure, stochasticity and increased population sizes especially in councils with small populations and low transmission intensities. With strata and intervention specific characteristics requiring additional parameters, one large simulation-model becomes inefficient and insufficient to address all programmatic questions. However, to allow for a constant re-evaluation and adjustments in interventions, a fast and flexible modelling approach using a parsimonious model that can be easily updated would be an advantage.

Third, while the results include heterogeneity within strata, the model results are likely less accurate at council level because seasonality, entomology as well as historical intervention parameters were assumed to be homogeneous for councils within a region. Sub-region as well as sub-council heterogeneity are increasingly relevant factors, as risk stratifications are being developed at higher resolution [81] and interventions will be deployed at finer spatial scale. These factors will also be increasingly feasible to account for in future models, as data quantity and quality, especially in routine data, as well as modelling capacities continue to improve. Similarly, urban and rural councils only differed in their transmission intensity and setting specific parameters for seasonality and intervention coverage, whereas socio-demographic factors [82], population density [83], local environment and infrastructure [84], as well as human mobility malaria case importation rates [85, 86] among other factors that are important considerations for intervention implementation were not accounted for.

Finally, the applied model primarily addresses the technical feasibility, not the operational or financial feasibility [87], hence model predictions are likely overestimated and should be interpreted in relative terms. The model predictions obtained in this analysis were designed for comparing the relative impact of the intervention mixes of interest at the time of the analysis rather than making predictions about the future impact of the strategy. This distinction is especially important since long-term temporal effects might bias the intervention impact predictions. Examples for long-term temporal effects include inter-annual variations in climate, that might lead to local malaria epidemics [88–91], or public health emergencies, such as the COVID-19 pandemic in 2020 [92] that caused service delivery interruptions and delays in several countries and was predicted to potentially substantially increase malaria burden and deaths [60, 93–95].

In mainland Tanzania, the IRS campaign in few councils got delayed by around six months due to COVID-19, while the ITN campaign initially planned for 2019, was rescheduled to 2020 due to reasons not related to COVID-19. Recent routine health facility data trends indicate a reduction in outpatient health care seeking and testing whereas test positivity ratio and incidence remained relatively constant. The next round of DHS has not been completed at the time of writing the manuscript and a validation of modelling results was not possible and would have been challenging due to the reasons outlined above. It is however critical to update and recalibrate these models as a dynamic process as new data becomes available to maintain an up-to date country specific model.

The results of this work extend previous work where modelling was applied to assess the technical feasibility of reaching the 2015–2020 NMSP target prevalence of less than 1% by 2020 and to explore alternative intervention allocations at council level that would lead to most impact on prevalence or be most cost-effective in reducing incidence [18]. In contrast to these previous objectives where modelling was used to obtain a new council stratification based on modelled impact, this work used modelling to obtain a comparison for selected interventions for a fixed council stratification based on malaria risk [16, 20]. Both use cases demonstrate the potential value of modelling to support the development of malaria strategic plans, which under the HBHI initiative [4] finds increasing application in other high burden countries [96].

In Tanzania, the shift from almost fully unconstrained mathematical modelling analysis (free combination of interventions and allocation to councils [18]) to a modelling analysis under meaningful constraints set by the NMCP (present analysis) demonstrates two distinct yet related use cases for applying modelling to inform a national malaria control strategy. Although risk stratification should be based on local data, geographical patterns in intervention impact predictions could play a supportive role for sub-national tailoring of interventions to guide intervention allocation for each risk stratum.

The process of subnational intervention impact modeling to support NMCPs will become more reliable and dynamic with the use of increasing high-quality routine data as basis for stratification and modelling. The increased use of mathematical modelling outputs in consultation with NMCPs will result in strengthened strategic and operational planning that will lead towards burden reduction and ultimately elimination.

## Conclusion

A modelling approach was presented for predicting the impact of intervention mixes targeted to malaria risk strata in mainland Tanzania as defined by the NMCP during the strategic planning process. By using a previously calibrated model, the model could readily address emerging questions and provided a powerful analytical insight into likely trends of intervention impacts on malaria prevalence and incidence across and within malaria risk strata. The application of modelling for exploring alternative intervention scenarios is likely to increase confidence in the selection of intervention mixes when developing a new national malaria control strategy. Continuous model updates and improvements in the approach will be crucial when scaling up the application of modelling for strategic planning processes in countries.

## Supplementary Information

**Additional file 1.** Strategic planning questions for modelling in mainland Tanzania.

**Additional file 2.** Historical trends per strata and fitting performance.

**Additional file 3.** Results table as included in the revised strategic plan.

## Data Availability

The simulation datasets generated and analysed during the current study are available in the Zenodo repository (https://doi.org/10.5281/zenodo.5701550), and analysis script available in the GitHub repository, https://github.com/ManuelaRunge/tza_nmsp2018_modeling. All other data are available in the paper and additional information files.

## Abbreviations

ACT: Artemisinin-based combination therapy
CM: Case management
EIR: Entomological Inoculation Rate
HBHI: High Burden to High Impact
ibppa: Infectious bites per person per annum
iCCM: Integrated Community Case Management
IPTp: Intermittent preventive therapy in pregnancy
IPTsc: Intermittent preventive therapy in school children
IRS: Indoor residual spraying
ITN: Insecticide-treated bed nets
LSM: Larval source management
MoHCDGEC: Ministry of Health, Community Development, Gender, Elderly and Children
MDA: Mass drug administration
MRC: Mass replacement campaigns
NMCP: National Malaria Control Programme
NMSP: National Malaria Strategic Plan
*Pf*PR: *Plasmodium falciparum* Parasite rate
SNP: School net programmes
SMC: Seasonal Malaria Chemoprevention
