# Supplementary material for: Sub-national tailoring of malaria interventions in Mainland Tanzania: simulation of the impact of strata-specific intervention combinations using modelling
Source: Malar J. 2022 Mar 17;21:92. doi: 10.1186/s12936-022-04099-5 (PMC8929286; doi:10.1186/s12936-022-04099-5)
Supplement: Supplementary file 1 — Additional file 1. Strategic planning questions for modelling in mainland Tanzania. [file 12936_2022_4099_MOESM1_ESM.docx]

# Additional file 1: Strategic planning questions for modelling in mainland Tanzania

**Explanation of past impact**

1. How could the increase in malaria prevalence since Tanzania HIV/AIDS and Malaria Indicator Survey 2012 (SMPS data) be explained/be predicted?

**Assessment of the feasibility of targets**

1. Will the proposed strategies in the Malaria Strategic Plan 2015 -2020 result in the target of <1% prevalence by 2020 in all parts of the country?

**Comparison of strategies**

1. What is the impact of revised versus current NMSP?
2. What are the costs per strata per intervention of the revised NMSP?
3. What are the differences in impact and costs of current and revised NMSP?

**Comparison of intervention combinations by setting**

1. What is the additional impact of IRS in combination to LLIN in settings where there is pyrethroid resistance?
2. Understanding the additional benefit of using PBO nets.
3. What is the additional impact of MDA in very low transmission districts (in low strata)
4. What is the impact of increased surveillance and active case detection system to target efforts for focal and reactive MDA

**Effectiveness of single interventions**

1. What is the difference in the impact of using ACT versus primaquine for MDA?
2. How sustainable is MDA, and what are the influencing factors?
3. What is the potential role of larviciding in urban areas and low transmission settings?
4. Does changing insecticide used for IRS to SumiShield lead to higher impact?

**Targeting of interventions**

1. What is the effect of adding PBO-nets through continuous distribution channels in regions with high insecticide resistance?
2. Should urban areas be handled differently when targeting interventions? (e.g. discontinuing mass LLIN distribution campaigns?)
3. Are ITNs still needed at persistent very low transmission?
4. Is CM enough to maintain low prevalence at persistent very low transmission?
5. Additional impact and costs of IPTsc (which is a not yet WHO recommended intervention) in high strata, only in high strata or also in moderate strata?

**Deployment and operational considerations**

1. What is the effect of alternative health care delivery modes for hard-to-reach populations?
2. What is the effect of scaling up diagnostics in the informal health sector (ADDO) and increasing access to affordable quality-assured ACT in the private sector?
3. What is the optimal deployment regimen for larviciding?
4. How to sequentially best deploy PBO nets? At which rate and how many districts to include?

**Technical questions**

1. How far to go back in time to produce accurate future predictions?
2. Which parameters to fit and which to fix?
3. How detailed do the historical interventions be included? (e.g. only considering ITN coverage, or also IRS and LSM?)
4. How detailed do the simulations need to be for district predictions? Generic simulations, simulations confined to administrative boundaries?
