# Supplementary material for: Sub-national tailoring of malaria interventions in Mainland Tanzania: simulation of the impact of strata-specific intervention combinations using modelling
Source: Malar J. 2022 Mar 17;21:92. doi: 10.1186/s12936-022-04099-5 (PMC8929286; doi:10.1186/s12936-022-04099-5)
Supplement: Supplementary file 2 — Additional file 2. Historical trends per strata and fitting performance. [file 12936_2022_4099_MOESM2_ESM.docx]

**Additional file 2: Historical trends per strata and fitting performance**

**Historical trend in prevalence**


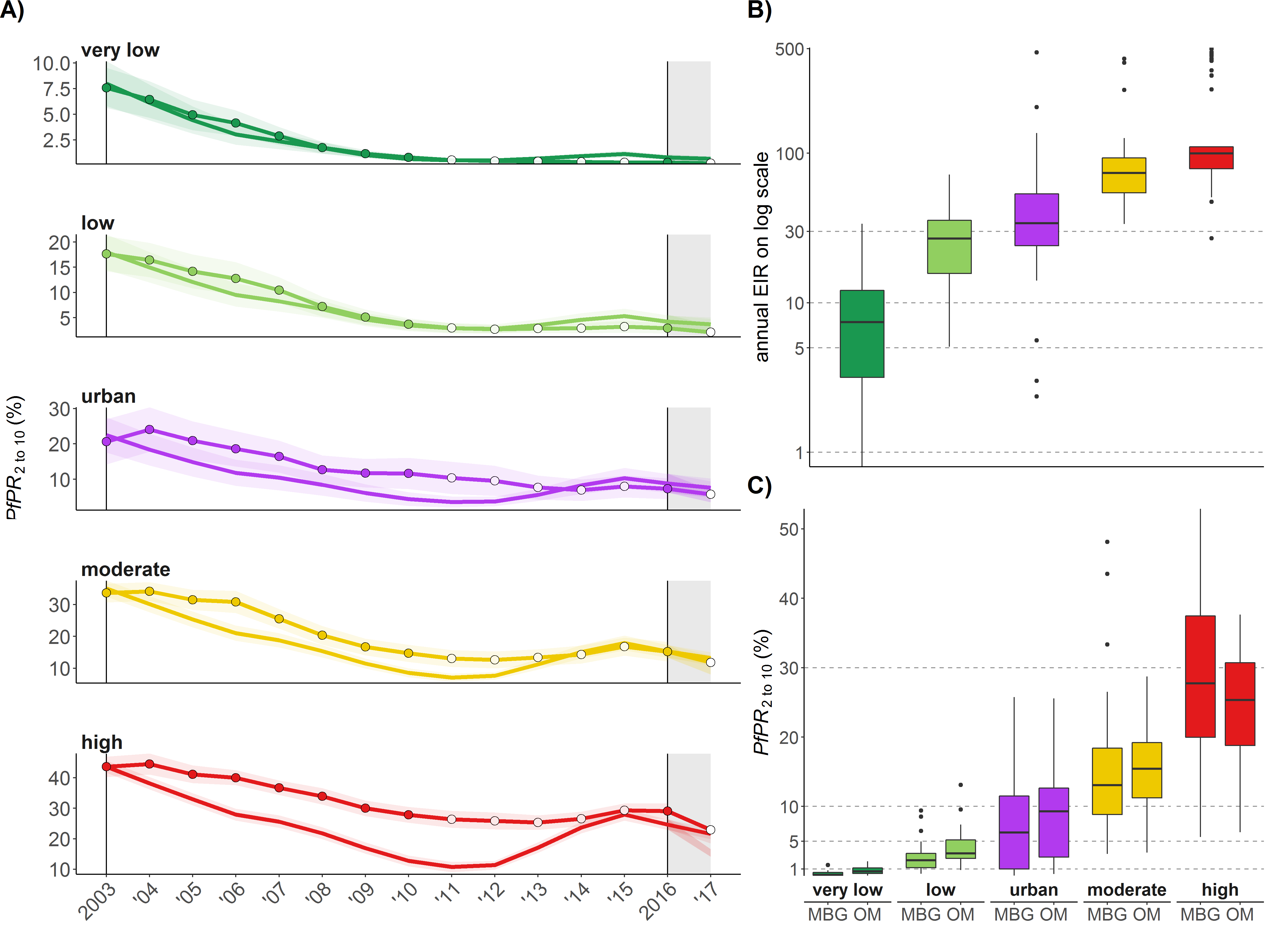


**Figure A2.1: Model-predicted and simulated malaria prevalence and estimated transmission intensity**

**A)** The lines without points show the prevalence simulated from the mathematical model and the line with annual points shows the geospatial predicted prevalence. The filled points indicate the estimates included and the white points the estimates not included in the fitting. The shaded area shows the confidence interval based on geographical heterogeneity between the councils. The vertical dashed lines show the baseline year and the grey shaded area the time between 2016 and 2017. **B)** Estimated pre-intervention EIR. **C)** Average-weighted simulated prevalence (OpenMalaria) in comparison to model-based geostatistical (MBG)-predicted prevalence for 2016.


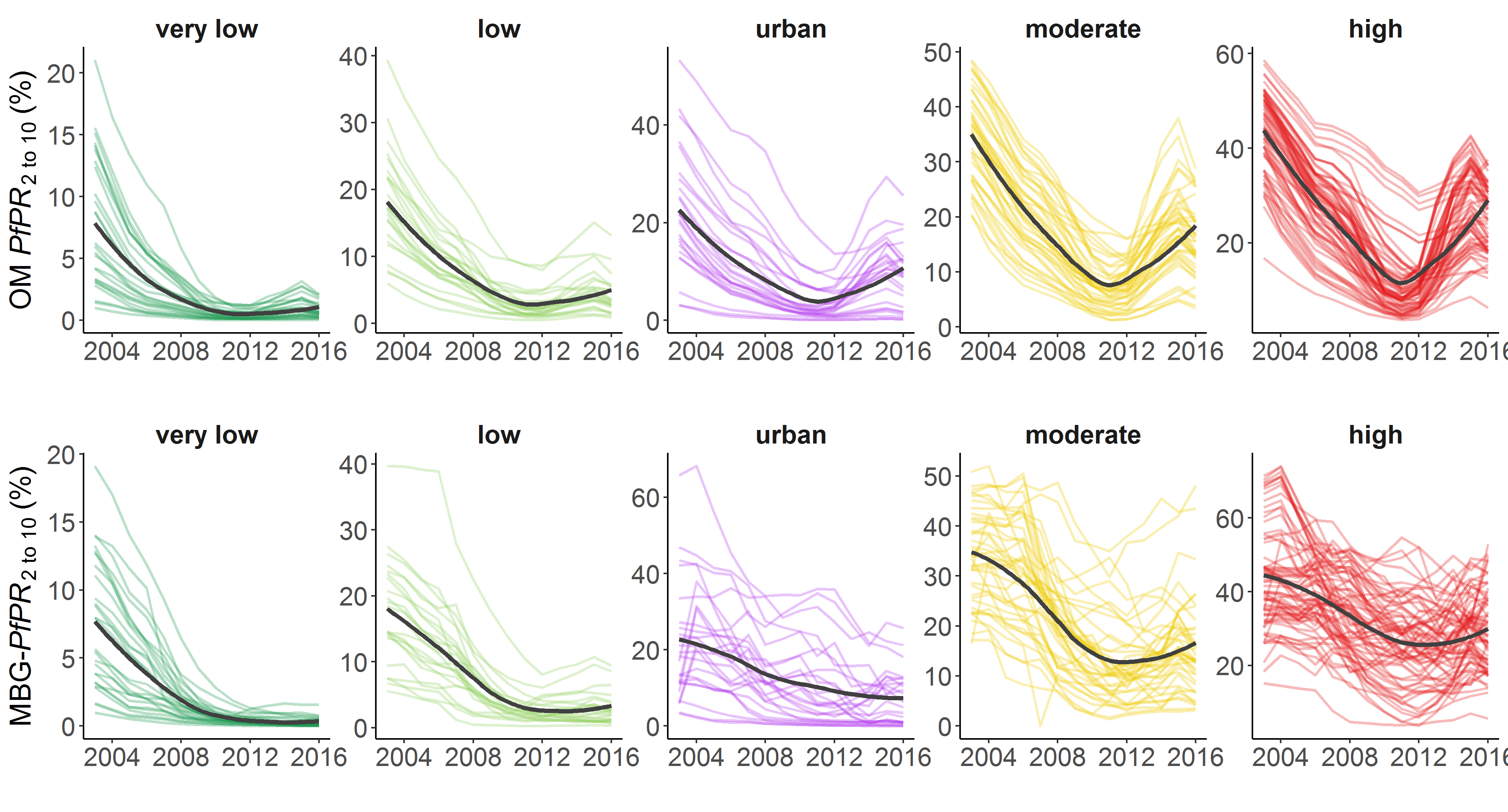


**Figure A2.2: Heterogeneity in malaria prevalence per council, grouped by strata, 2003-2016**

The historical trend in simulated prevalence (top row) and geospatial predicted prevalence (bottom row). Each line corresponds to one council and the black line shows the average per strata.


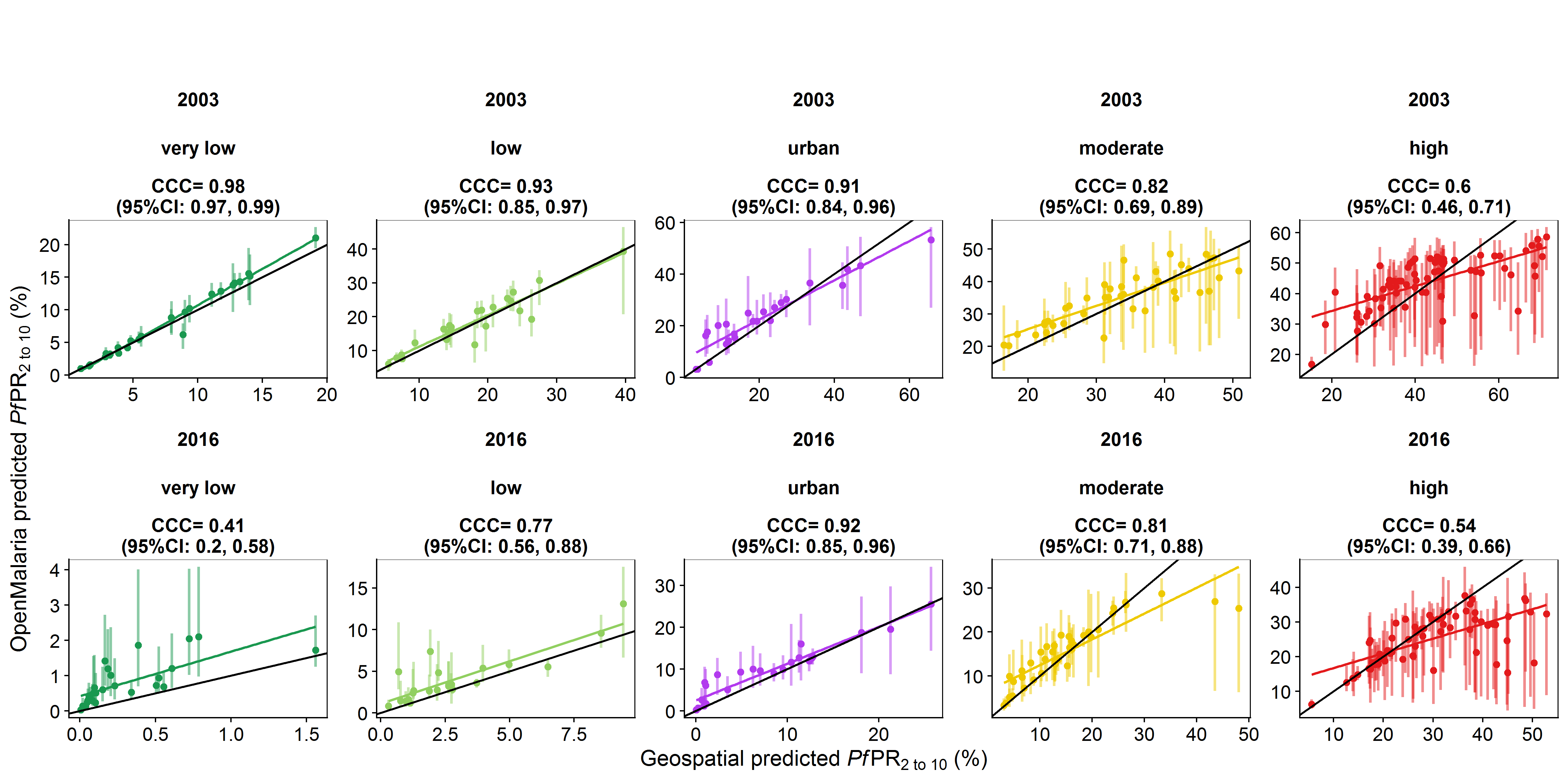


**Figure A2.3: Fitting performance for simulated prevalence per council grouped by strata**

The figure shows the predicted prevalence and the geo-statistical model prevalence. The pre-intervention (2003) and baseline year (2016) are the most relevant historical time points since the prevalence before the deployment of interventions determines the level of possible rebound, and the prevalence in the baseline year is used as a comparator for assessing the relative impact of future interventions. The scatter plots shows the respective prevalence estimates with the regression line (coloured line), and perfect correspondence line (black line) grouped by strata. CCC = Lin’s concordance correlation coefficients^[[1]](#footnote-1)^.

**Future intervention predictions**


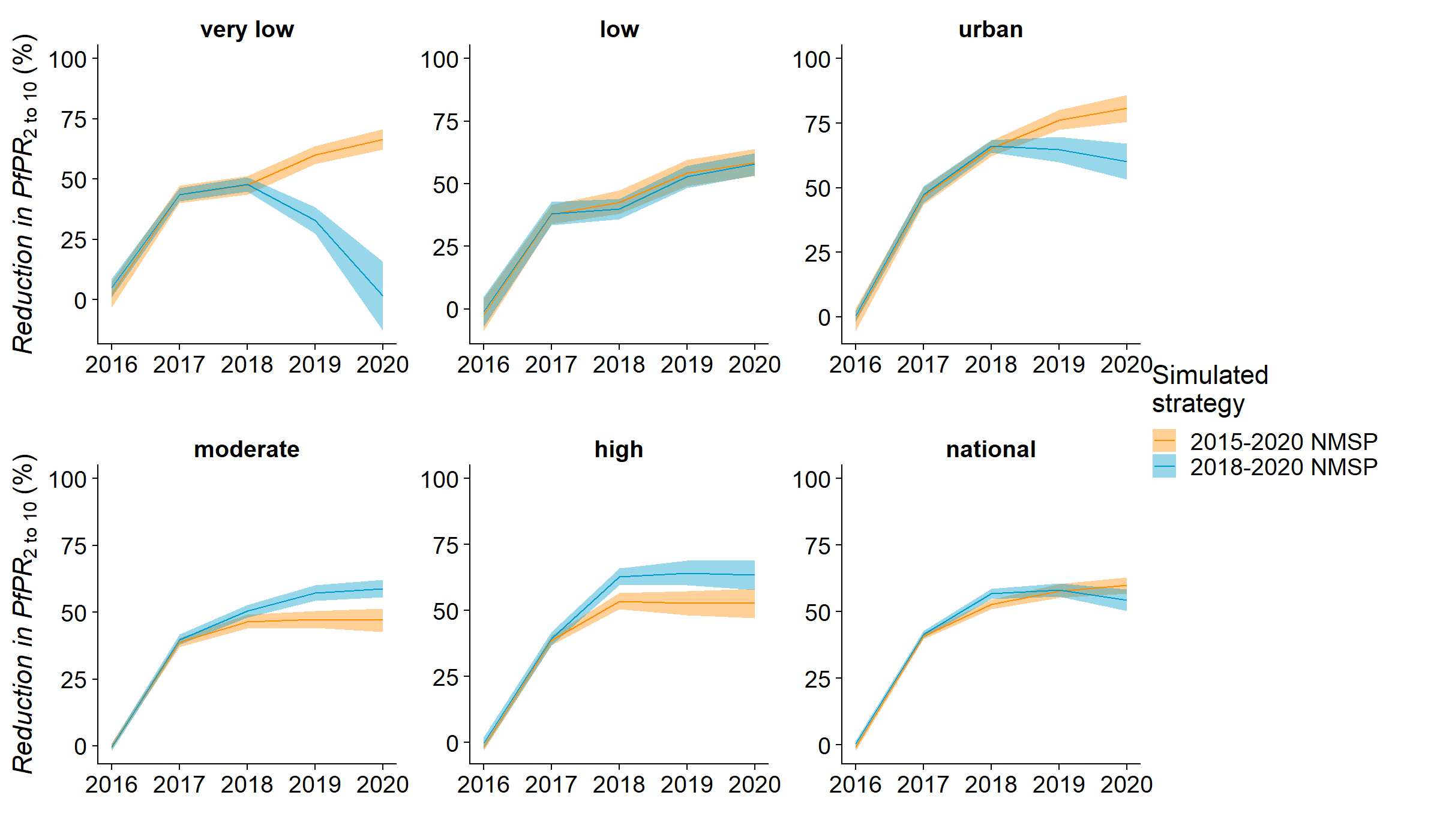


**Figure A2.4:** **Predicted prevalence reduction between 2016 and 2020 per strata and strategy**

Using population-weighted mean and 95%CI

**Table A2.1: Predicted reduction in prevalence in 2020 compared to 2016 per strata and strategy**

|  |  | **mean** | | **lower.ci** | | **upper.ci** | |
| --- | --- | --- | --- | --- | --- | --- | --- |
| **Strata** | **NMSP** | **w** | **unw** | **w** | **unw** | **w** | **unw** |
| very low | 2015-2020 NMSP | 66.5 | 66.1 | 62.2 | 61.8 | 70.8 | 70.3 |
| very low | 2018-2020 NMSP | 1.5 | -5.4 | -12.8 | -21.2 | 15.8 | 10.5 |
| low | 2015-2020 NMSP | 58.5 | 56.2 | 53 | 50.5 | 63.9 | 61.9 |
| low | 2018-2020 NMSP | 57.8 | 57.2 | 53.4 | 52.4 | 62.3 | 62 |
| urban | 2015-2020 NMSP | 80.7 | 73.5 | 75.5 | 65.4 | 86 | 81.6 |
| urban | 2018-2020 NMSP | 60.1 | 67.7 | 53.1 | 59.6 | 67 | 75.7 |
| moderate | 2015-2020 NMSP | 47.1 | 48.7 | 42.7 | 43.8 | 51.4 | 53.7 |
| moderate | 2018-2020 NMSP | 58.9 | 59.5 | 55.7 | 55.4 | 62.1 | 63.6 |
| high | 2015-2020 NMSP | 52.8 | 47.4 | 47.2 | 41 | 58.4 | 53.8 |
| high | 2018-2020 NMSP | 63.5 | 57.5 | 57.9 | 51.1 | 69.2 | 63.9 |
| national | 2015-2020 NMSP | 59.9 | 55 | 56.9 | 51.7 | 63 | 58.3 |
| national | 2018-2020 NMSP | 54.3 | 50.4 | 50.3 | 45.6 | 58.4 | 55.2 |

w=population weighted summary statistics, unw=unweighted summary statistics

**Table A2.2: Predicted proportion of the population per prevalence category in 2020**

| **Prevalence**  **categories (%)** | **Proportion of the population (%)** | |
| --- | --- | --- |
|  | **2015-2020 NMSP** | **2018-2020 NMSP** |
| <=1 | 24.7 | 13.4 |
| >1-5 | 23.6 | 29.5 |
| >5-10 | 31.1 | 47.5 |
| >10-25 | 19 | 8.7 |
| >25-50 | 1.7 | 1.0 |
| Total | 100 | 100 |


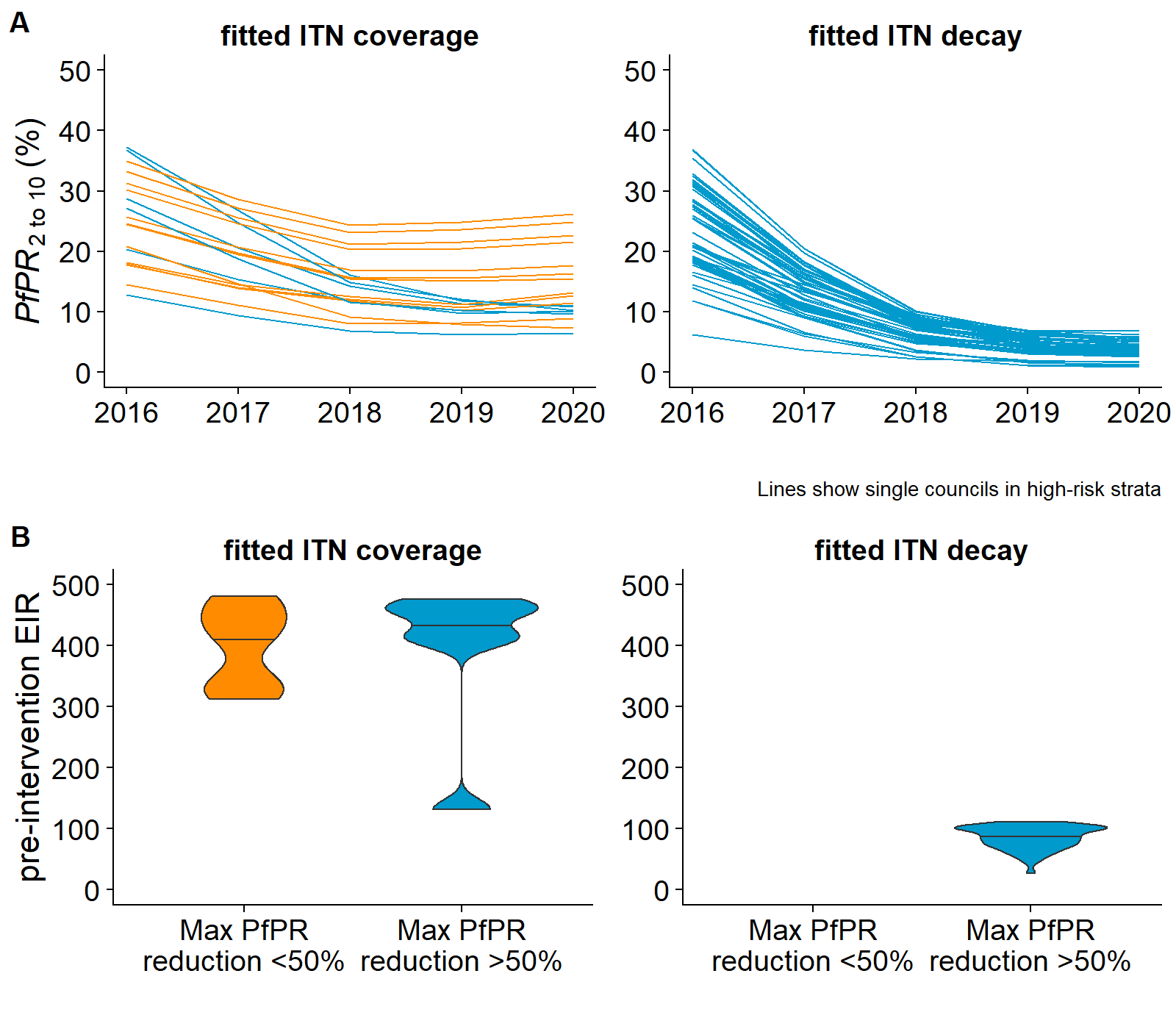


**Figure A2.5: Predicted prevalence trend and pre-intervention EIR for councils in the high region grouped by ITN parameter used in model fitting and predicted prevalence reduction.** The orange colour highlights 13 councils in which the maximum prevalence reduction between 2016 and 2020 was less than 50% even when combining all interventions. For high-risk councils that received a school campaign (in Mtwara, Ruvuma, Lindi region, Southern Tanzania), the ITN coverage between 2012 and 2016 was fitted compared to the ITN decay. These councils were also the concils with highest transmission (see Runge et al 2020^[[2]](#footnote-2)^ for detailed description of model calibration).

1. Lin LI-K. A Concordance Correlation Coefficient to Evaluate Reproducibility. Biometrics. 1989;45: 255–268. doi:10.2307/2532051 [↑](#footnote-ref-1)
2. Runge, M., Snow, R.W., Molteni, F., Thawer, S., Mohamed, A., Mandike, R., Giorgi, E., Macharia, P.M., Smith, T.A., Lengeler, C., Pothin, E., 2020b. Simulating the council-specific impact of anti-malaria interventions: A tool to support malaria strategic planning in Tanzania. PloS One 15, e0228469. [↑](#footnote-ref-2)
