# Supplementary material for: Sub-national tailoring of malaria interventions in Mainland Tanzania: simulation of the impact of strata-specific intervention combinations using modelling
Source: Malar J. 2022 Mar 17;21:92. doi: 10.1186/s12936-022-04099-5 (PMC8929286; doi:10.1186/s12936-022-04099-5)
Supplement: Supplementary file 3 — Additional file 3. Results table as included in the revised strategic plan. [file 12936_2022_4099_MOESM3_ESM.docx]

**Table A3.1: Results table as included in the revised strategic plan***

| **Stratum** | **Modelling Results** | **Strategic response and mitigation** |
| --- | --- | --- |
| **Very low** | - Without ITN replacements but with high effective treatment rate, the prevalence was predicted to be maintained until 2020. - Depending on the pre-intervention EIR, a slight increase in prevalence was predicted after the effect of the last MRC (2016) decayed, while prevalence in 2020 remained lower as the baseline prevalence in 2016. | - Establishment of improved surveillance and response, including case-based surveillance with reactive case detection; Foci investigation and response with reactive vector control interventions; Epidemic detection and response |
| **Low** | - LLINs were assumed to be maintained at least 40% coverage in the population (e.g. through continuous distributions) with improved CM at least 80% and results predicted to maintain the baseline prevalence in 2016 until 2020. - In the scenario without additional implementation of ITNs, the prevalence was predicted to increase in 2020. | - Establishment of ITN coverage surveillance for optimizing ITN distribution mechanisms. - Improved malaria surveillance and response including epidemic detection and response |
| **Moderate** | - ITN mass campaign followed by continuous ITN distributions (e.g. SNP), was predicted to result in a large decrease in prevalence until 2020 if no other control measure was in place. | - Establishment of ITN coverage surveillance for optimizing ITN distribution mechanisms. |
| **High** | - The combination of annual ITN distributions, maintaining an ITN coverage of at least 70%, additional IRS campaigns in districts in the Lake Zone and Kigoma, and IPTsc in all districts was predicted to result in high reductions in prevalence until 2020, which reached the same predicted prevalence as in the moderate strata. - Implementation of IPTsc showed some additional impact and the deployment would need to be assessed at the council level. - The combination of mass campaign and annual ITN distributions led to additional marginal reductions. | - Establishment of ITN coverage surveillance for optimizing ITN distribution mechanisms. - IRS to be deployed according to optimized resource allocation. - Micro-stratification to detect possible candidate areas for IPTsc |
| **Urban** | - The baseline prevalence in 2016 was highly heterogeneous, and discontinuation of ITN distributions in all urban areas was predicted to result in a rebound in prevalence. - Therefore, ITN distributions in urban areas should follow epidemiological strata. - In practice, LSM, e.g. larviciding, might have an additional impact but was excluded from the analysis. | - ITN distribution mechanisms to be deployed according to the underlying epidemiological stratum. - Optimal CM coverage to be reached with private sector quality improvement schemes. |

*) edited and shortened version from the SMMSP^[[1]](#footnote-1)^.


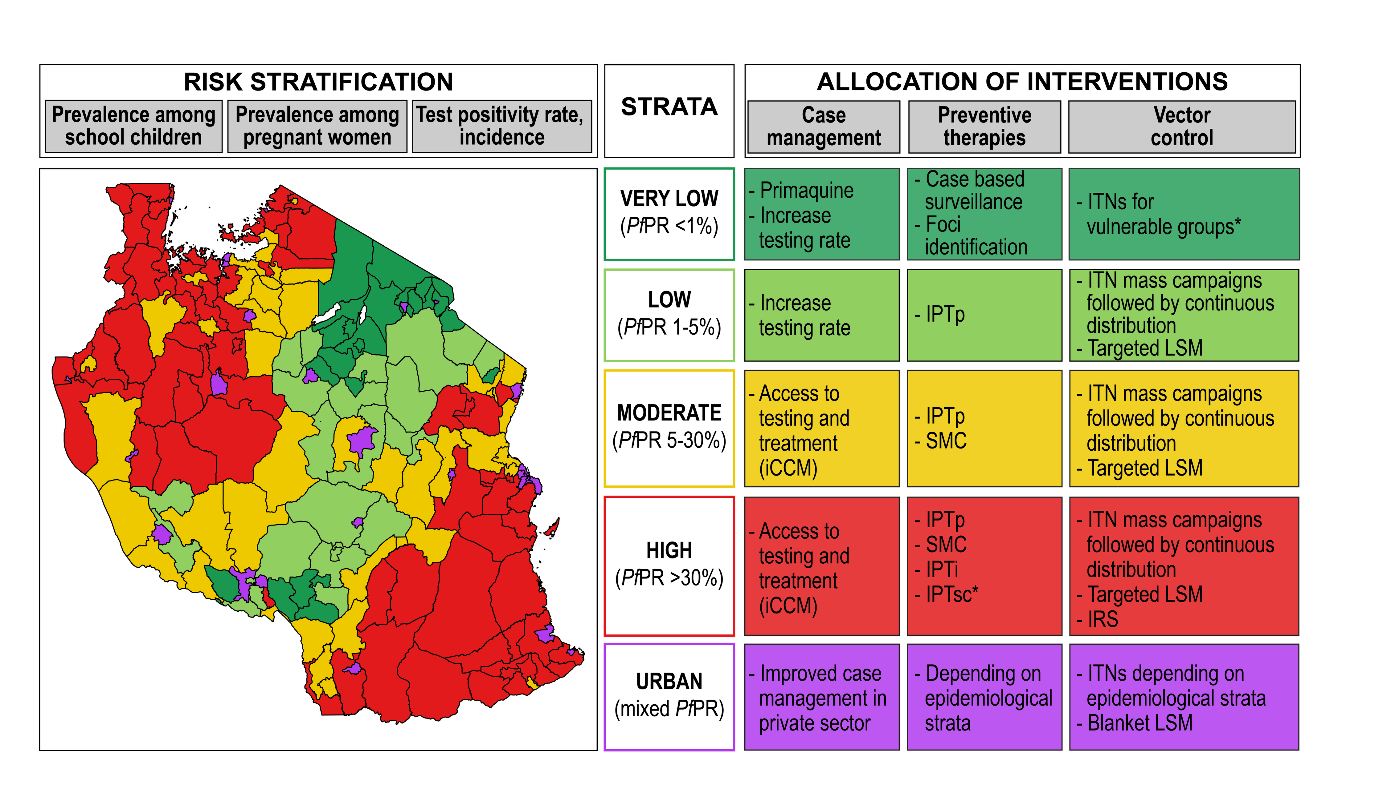


**Fig A3.1: Overview of malaria risk stratification and selected interventions per strata in Mainland Tanzania**

This figure is a simplified version, adapted from the supplementary malaria midterm strategic plan (2018-2020 NMSP). that includes details on the stratification method^[[2]](#footnote-2)^, intervention deployment and antimalarial drugs used. **Additional intervention allocation constraints and sub-groups:** IRS regions were Kagera, Geita, Mara, Mwanza, and Kigoma. Councils eligible for SMC included Nachingwea DC, Bahi DC, Itigi DC, Nanyumbu DC, Masasi DC, and Manyoni DC. Continuous ITN distribution through schools (ITN-SNP) was operational in fourteen regions (Katavi, Kagera, Mara, Mwanza, Simiyu, Shinyanga, Geita, Lindi, Mtwara, Ruvuma, Morogoro, Tabora, Kigoma, Pwani), and in those areas no ITN-MRC was planned for 2019.

***)** Interventions not included in the WHO recommendations in 2018. In the very low strata, ITNs were considered for vulnerable groups only while universal coverage is recommended for ITNs in all malaria endemic areas^[[3]](#footnote-3)^.

**Abbreviations**: CM: Case management; iCCM= integrated Community Case Management; ITN=Insecticide Treated Nets; IPT=Intermittent preventive therapy in (p) Pregnancy, (i) infants, (sc) school children; IRS: Indoor Residual Spraying. LLIN: Long-lasting insecticide treated nets. LSM= Larval Source Management. SMC: Seasonal Malaria Chemoprevention. SMMSP: Supplementary malaria midterm strategic plan.

1. MoHCDGEC. Supplementary malaria midterm strategic plan 2018-2020. Dar Es Salaam, Tanzania: Ministry of Health Community Development Gender Elderly & Children (MoHCDGEC). National Malaria Control Programme (NMCP).; 2019 Sep [↑](#footnote-ref-1)
2. Thawer SG, Chacky F, Runge M, Reaves E, Mandike R, Lazaro S, et al. Sub-national stratification of malaria risk in mainland Tanzania: a simplified assembly of survey and routine data. Malar J. 2020;19: 177. [↑](#footnote-ref-2)
3. WHO. Guidelines for malaria vector control. Geneva, Switzerland: World Health Organization (WHO); 2019. [↑](#footnote-ref-3)
